# ACE-SNN: Algorithm-Hardware Co-design of Energy-Efficient & Low-Latency Deep Spiking Neural Networks for 3D Image Recognition

**DOI:** 10.3389/fnins.2022.815258

**Published:** 2022-04-07

**Authors:** Gourav Datta, Souvik Kundu, Akhilesh R. Jaiswal, Peter A. Beerel

**Affiliations:** Ming Hsieh Department of Electrical and Computer Engineering, University of Southern California, Los Angeles, CA, United States

**Keywords:** hyperspectral images, spiking neural networks, quantization-aware, gradient descent, processing-in-memory

## Abstract

High-quality 3D image recognition is an important component of many vision and robotics systems. However, the accurate processing of these images requires the use of compute-expensive 3D Convolutional Neural Networks (CNNs). To address this challenge, we propose the use of Spiking Neural Networks (SNNs) that are generated from iso-architecture CNNs and trained with quantization-aware gradient descent to optimize their weights, membrane leak, and firing thresholds. During both training and inference, the analog pixel values of a 3D image are directly applied to the input layer of the SNN without the need to convert to a spike-train. This significantly reduces the training and inference latency and results in high degree of activation sparsity, which yields significant improvements in computational efficiency. However, this introduces energy-hungry digital multiplications in the first layer of our models, which we propose to mitigate using a processing-in-memory (PIM) architecture. To evaluate our proposal, we propose a 3D and a 3D/2D hybrid SNN-compatible convolutional architecture and choose hyperspectral imaging (HSI) as an application for 3D image recognition. We achieve overall test accuracy of 98.68, 99.50, and 97.95% with 5 time steps (inference latency) and 6-bit weight quantization on the Indian Pines, Pavia University, and Salinas Scene datasets, respectively. In particular, our models implemented using standard digital hardware achieved accuracies similar to state-of-the-art (SOTA) with ~560.6× and ~44.8× less average energy than an iso-architecture full-precision and 6-bit quantized CNN, respectively. Adopting the PIM architecture in the first layer, further improves the average energy, delay, and energy-delay-product (EDP) by 30, 7, and 38%, respectively.

## 1. Introduction

3D image classification is an important problem, with applications ranging from autonomous drones to augmented reality. 3D content creation has been gaining momentum in the recent past and the amount of information in the form of 3D input data becoming publicly available is steadily increasing. In particular, hyperspectral imaging (HSI), which extracts rich spatial-spectral information about the ground surface, has shown immense promise in remote sensing (Chen et al., 2014), and thus, has become an important application for 3D image recognition. HSI is currently used in several workloads ranging from geological surveys (Wan et al., 2021), to the detection of camouflaged vehicles (Papp et al., 2020). In hyperspectral images (HSIs), each pixel can be modeled as a high-dimensional vector where each entry corresponds to the spectral reflectivity of a particular wavelength (Chen et al., 2014), and constitutes the 3^*rd*^ dimension of the image. The goal of the classification task is to assign a unique semantic label to each pixel (Zheng et al., 2020). For HSI classification, several spectral feature-based methods have been proposed, including support vector machine (Melgani and Bruzzone, 2004), random forest (Pal, 2003), canonical correlation forest (Xia et al., 2017), and multinomial logistic regression (Krishnapuram et al., 2005). However, these spectral-spatial feature extraction methods rely on hand-designed descriptions, prior information, and empirical hyperparameters (Chen et al., 2014).

Lately, convolutional neural networks (CNNs), consisting of a series of hierarchical filtering layers for global optimization have yielded higher accuracy than the hand-designed features (Krizhevsky, 2012), and have shown promise in multiple applications including image classification (He et al., 2016), object detection (Ren et al., 2017), semantic segmentation (He et al., 2018), and depth estimation (Repala and Dubey, 2019). The 2D CNN stacked autoencoder (Chen et al., 2014) was the first attempt to extract deep features from its compressed latent space to classify HSIs. To extract the spatial-spectral features jointly from the raw HSI, researchers proposed a 3D CNN architecture (Ben Hamida et al., 2018), which achieved SOTA classification results. In Lee and Kwon (2017), Roy et al. (2020), and Luo et al. (2018) successfully created multiscale spatiospectral relationships using 3D CNNs and fused the features using a 2D CNN to extract more robust representation of spectral–spatial information. However, compared to 2D CNNs used to classify traditional RGB images, multi-layer 3D CNNs require significantly higher power and energy costs (Li et al., 2016). A typical hyperspectral image cube consists of several hundred spectral frequency bands that, for target tracking and identification, require real time on-device processing (Hien Van Nguyen et al., 2010). This desire for HSI sensors operating on energy-limited devices motivates exploring alternative lightweight classification models.

**Figure 1:**
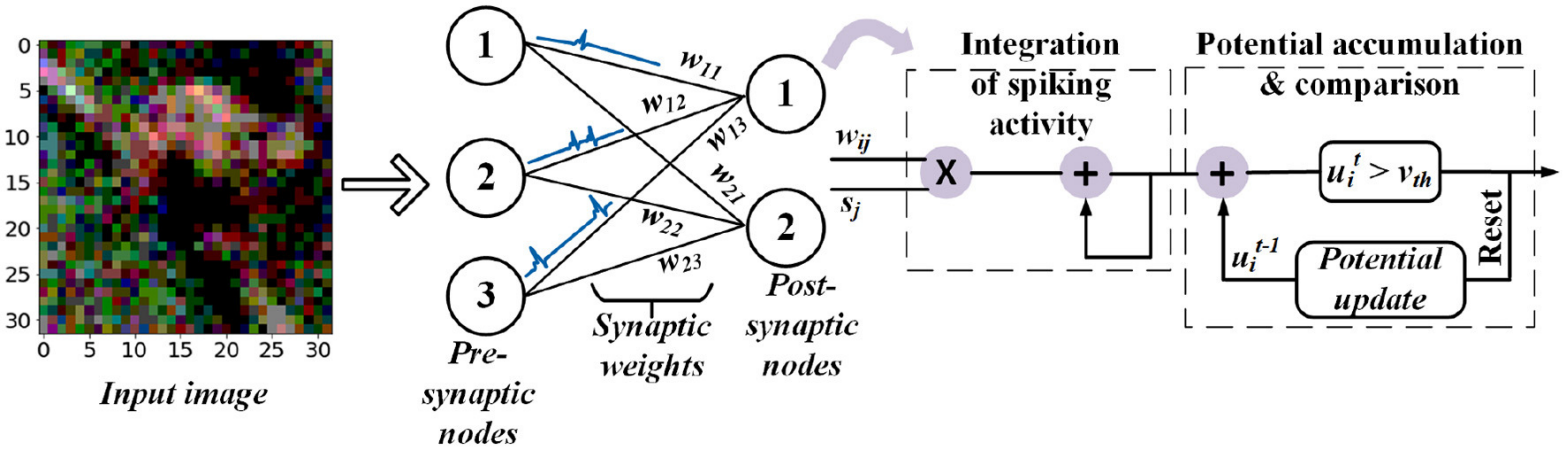
Feedforward fully-connected SNN architecture with integrate and fire (IF) spiking dynamics.

In particular, low-latency spiking neural networks (SNNs) (Pfeiffer and Pfeil, 2018), illustrated in Figure 1, have gained attention because they are more computational efficient than CNNs for a variety of applications, including image analysis. To achieve this goal, analog inputs are first encoded into a sequence of spikes using one of a variety of proposed encoding methods, including rate coding (Diehl et al., 2016; Sengupta et al., 2019), direct coding (Rathi and Roy, 2020), temporal coding (Comsa et al., 2020), rank-order coding (Kheradpisheh and Masquelier, 2020), phase coding (Kim et al., 2018), and other exotic coding schemes (Almomani et al., 2019; Datta et al., 2021). Among these, direct coding have shown competitive performance on complex tasks (Diehl et al., 2016; Sengupta et al., 2019) while others are either limited to simpler tasks such as learning the XOR function and classifying MNIST images or require a large number of spikes for inference.

In addition to accommodating various forms of encoding inputs, supervised learning algorithms for SNNs have overcome various roadblocks associated with the discontinuous derivative of the spike activation function (Lee et al., 2016; Wu et al., 2019). In particular, recent works have shown that SNNs can be efficiently converted from artifical neural networks (ANNs) by approximating the activation value of ReLU neurons with the firing rate of spiking neurons (Sengupta et al., 2019). Low-latency SNNs trained using ANN-SNN conversion, coupled with supervised training, have been able to perform at par with ANNs in terms of classification accuracy in traditional image classification tasks (Rathi and Roy, 2020; Datta and Beerel, 2021; Kundu et al., 2021c). Consequently, SNNs have lower compute cost than their non-spiking CNN counterparts. This is particularly useful in 3D CNNs which have higher arithmetic intensity (the ratio of floating point operations to accessed bytes) than 2D CNNs. This motivates this work which explores the effectiveness of SNNs converted from 3D CNNs for HSI classification.

To improve energy efficiency, model compression techniques, such as pruning (Han et al., 2015a), can be adapted to CNN/SNN models for HSI classification. Unstructured pruning can lead to significant parameter reduction (>10× for both 2D CNN and SNN models for traditional vision tasks (Kundu et al., 2021a,b). However, unstructured pruning typically requires specialized ASIC/FPGA hardware to reap energy-savings benefits and does not lead to savings when implemented on standard GPUs. On the other hand, structured pruning is compatible with general-purpose CPU/GPU hardware, but is unable to remove a large number of weights while maintaining accuracy, particularly for our proposed compact CNN architectures.

The energy efficiency of SNN inference can also be improved by using integer or fixed-point computational units implemented either as CMOS-based digital accumulators or memory array based processing-in-memory (PIM) accelerators. Previous research (Rathi et al., 2017; Sulaiman et al., 2020) have proposed post-training SNN quantization tailored toward unsupervised learning, which has not been shown to scale to complex vision tasks without requiring high precision (≥ 8 bits). This work addresses this gap by proposing a quantization-aware SNN training algorithm that requires only 5 time steps with 6-bit weights, yielding a 2× reduction in bit width compared to a post-training quantization baseline that yields similar accuracy.

The first layer in direct coded SNNs still requires multiply-and-accumulates (MAC), which are significantly more expensive than the accumulates required in a spiking layer. To mitigate this issue, we propose an SRAM-based processing-in-memory (PIM) architecture to process the first layer, which cannot only reduce the CMOS-based digital MAC cost, but also address the Von-Neumann bottleneck by eliminating data movement between the memory and the convolutional processing elements. Moreover, the relatively lower parameter count of the first 3D CNN layer ensures that we can perform the whole convolution in a single memory array, thereby improving area efficiency. The remaining layers, which involves cheap accumulates and threshold comparisons, are implemented with highly parallel programmable digital architectures, as used in Chen et al. (2022) and Park et al. (2019). Our proposed hardware-software co-design results in 700.7× and 1.79× improvements in energy-delay product (EDP) for HSI classification compared to digital implementatons with standard ANNs and SNNs.

In summary, this paper provides the following contributions:

- We analyse the arithmetic intensities of 3D and 2D CNNs, and motivate the use SNNs to address the compute energy bottleneck faced by 3D CNN layers used for HSI classification.
- We propose a hybrid training algorithm that first converts an ANN for HSI classification to an iso-architecture SNN, and then trains the latter using a novel quantization-aware spike timing dependent backpropagation (Q-STDB) algorithm that yields low latency (5 time steps) and low bit width (6-bits).
- We propose two compact convolutional architectures for HSI classification that can yield classification accuracies similar to state-of-the-art (SOTA) and are compatible with our ANN-SNN conversion framework.
- We propose a novel circuit framework and its associated energy models for energy-efficient hardware implementation of the SNNs obtained by our training framework, and benchmark the EDP gains compared to standard ANNs. Our experimental results reveal that the SNNs trained for HSI classification offer four and two orders of magnitude improvement in energy consumption compared to full-precision and iso-precision ANNs.

The remainder of this paper is structured as follows. In Section 2 we present necessary background and related work. Section 3 describes our analysis of arithmetic intensities of 3D and 2D CNNs, and highlights the motivation of using SNNs for 3D imaging. Sections 4, 5 discusses our proposed quantization-aware SNN training method and a PIM architecture to improve the energy efficiency of our proposed SNN models during inference. Section 6 focuses on our proposed network architectures, benchmark datasets, and our training details. We present detailed experimental results and analysis in Section 7. Finally, the paper concludes in Section 8.

## 2. Background

### 2.1. SNN Modeling

The spiking dynamics of a neuron are generally modeled using either the Integrate-and-Fire (IF) (Burkitt, 2006) or Leaky-Integrate-and-Fire (LIF) model (Lee et al., 2020), where the activity of pre-synaptic neurons modulates the membrane potential of postsynaptic neurons. The membrane potential of a IF neuron does not change during the time period between successive input spikes while in the LIF model, the membrane potential leaks at a constant rate. In this work, we adopt the LIF model in our proposed training technique, as the leak term improves the bio-plausibility and robustness to noisy spike-inputs (Chowdhury et al., 2020).

The LIF is probably one of the earliest and simplest spiking neuron models, but it is still very popular due to the ease with which it can be analyzed and simulated. In its simplest form, a neuron is modeled as a “leaky integrator” of its input *I*(*t*):


(1) $$\begin{array}{l} \tau_{m}\frac{\partial v}{\partial t}=-v\left(t\right)+R\cdot I\left(t\right) \end{array}$$

where *v*(*t*) represents the membrane potential of the neuron at time *t*, τ_*m*_ is the membrane time constant and *R* is the membrane resistance. When *v*(*t*) reaches a certain firing threshold, it is instantaneously reset to a lower value *v*_*r*_ (reset potential), the neuron generates a spike, and the leaky integration process described by Equation 1 starts afresh with the initial value *v*_*r*_. However, due to its continuous representation, Equation (1) is not suitable for implementations in popular Machine Learning (ML) frameworks (e.g., Pytorch). Hence, we convert Equation (1) into an iterative discrete-time version, as shown in Eqs. 2 and 3, within which spikes in a particular layer *l*, denoted as $o_{l}^{t}$, are characterized as binary values (1 represents the presence of a spike) (Rathi et al., 2020). The pre-spikes in the (*l* − 1)^*th*^ layer, $o_{l-1}^{t}$ are modulated by the synaptic weights ŵ_*l*_ to be integrated as the current influx in the membrane potential $u_{l}^{t}$ that decays with a leak factor λ_*l*_.


(2) $$\begin{array}{l} u_{l}^{t}=\lambda_{l}u_{l}^{t-1}+{ŵ}_{l}o_{l-1}^{t}-v_{l}o_{l}^{t} \end{array}$$

(3) $$\begin{array}{l} z_{l}^{t}=\frac{u_{l}^{t}}{v_{l}}-1,\;o_{l}^{t}=\{\begin{array}{ll} 1, & \text{if }z_{l}^{t}>0\\ 0, & \text{otherwise } \end{array} \end{array}$$

The third term in Equation (2) exhibits soft reset by setting the reset potential to the threshold *v*_*l*_ (instead of 0) i.e., reducing the membrane potential *u*_*l*_ by *v*_*l*_ at time step *t*, if an output spike is generated at the *t*^*th*^ time step. As shown in Rathi et al. (2020), soft reset enables each spiking neuron to carry forward the surplus potential above the firing threshold to the subsequent time step (Rathi et al., 2020), thereby minimizing the information loss.

### 2.2. SNN Training Techniques

Recent research on training supervised deep SNNs can be primarily divided into three categories: 1) ANN-SNN conversion-based training, 2) Spike timing dependent backpropagation (STDB), and 3) Hybrid training.

#### 2.2.1. ANN-SNN Conversion

ANN-SNN conversion involves copying the SNN weights from a pretrained ANN model and estimating the threshold values in each layer by approximating the activation value of ReLU neurons with the firing rate of spiking neurons (Cao et al., 2015; Diehl et al., 2015; Rueckauer et al., 2017; Hu et al., 2018; Sengupta et al., 2019). The ANN model is trained using standard gradient descent based methods and helps an iso-architecture SNN achieve impressive accuracy in image classification tasks (Rueckauer et al., 2017; Sengupta et al., 2019). However, the SNNs resulting from these conversion algorithms require an order of magnitude more time steps compared to other training techniques (Sengupta et al., 2019). In this work, we use ANN-SNN conversion as an initial step in Q-STDB because it is of relatively low complexity and yields high classification accuracy on deep networks as shown in Section 7.5.3.

#### 2.2.2. STDB

The thresholding-based activation function in the IF/LIF model is discontinuous and non-differentiable, which poses difficulty in training SNNs with gradient-descent based learning methods. Consequently, several approximate training methodologies have been proposed (Lee et al., 2016; Panda and Roy, 2016; Bellec et al., 2018; Neftci et al., 2019), where the spiking neuron functionality is either replaced with a differentiable model or the real gradients are approximated as surrogate gradients. However, the backpropagation step requires these gradients to be integrated over all the time steps required to train the SNN, which significantly increases the memory requirements.

#### 2.2.3. Hybrid Training

A recent paper (Rathi et al., 2020) proposed a hybrid training technique where the ANN-SNN conversion is performed as an initialization step and is followed by an approximate gradient descent algorithm. The authors observed that combining the two training techniques helps SNNs converge within a few epochs while requiring fewer time steps. In Rathi and Roy (2020) extended the above hybrid learning approach by training the membrane leak and the firing threshold along with other network parameters (weights) via gradient descent. Moreover, Rathi and Roy (2020) applied direct-input encoding where the pixel intensities of an image are applied into the SNN input layer as fixed multi-bit values each time step to reduce the number of time steps needed to achieve SOTA accuracy by an order of magnitude. Though the first layer now requires MACs, as opposed to cheaper accumulates in the remaining layers, the overhead is negligible for deep convolutional architectures (Rathi and Roy, 2020). This work extends these hybrid learning techniques by using a novel representation of weights for energy efficiency and performing quantization-aware training in the SNN domain.

## 3. 3D vs 2D CNNs: Arithmetic Intensity

**Figure 2:**
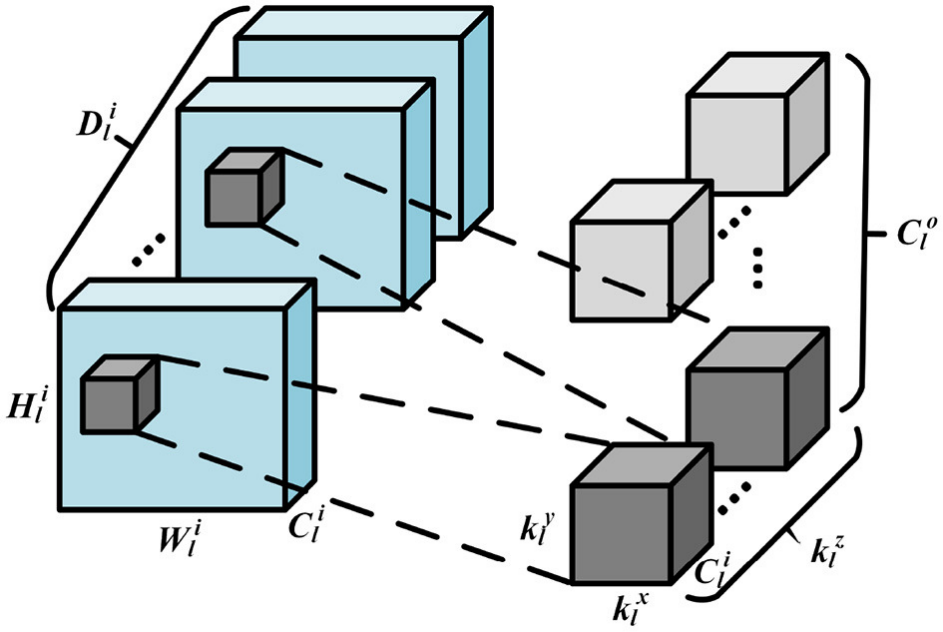
Illustration of the 3D convolution operation.

In this section, we motivate using SNNs to classify 3D images. As discussed earlier, 3D images require 3D convolutions to extract both coarse and fine-grained features from all three dimensions. Essentially, it's the same as 2D convolutions, but the kernel sliding is now 3-dimensional, enabling a better capture of dependencies within the 3 dimensions and creating a difference in output dimensions post convolution. The kernel of the 3D convolution will move in 3-dimensions if the kernel's depth is less than the feature map's depth. Please see the illustration in Figure 2, where width, height, and depth of a convolutional kernel are given by $k_{l}^{x}$, $k_{l}^{y}$, and $k_{l}^{z}$, respectively. $H_{l}^{i}$, $W_{l}^{i}$, and $D_{l}^{i}$ represents the height, weight, and depth for the input feature map. $C_{l}^{i}$ and $C_{l}^{o}$ denote the channel numbers of input and output feature map, respectively. Note that 3D convolutions are compute dominated, because the filters are strided in three directions for all the input channels to obtain a single output activation.

Let us evaluate the compute and memory access cost of a 3D CNN layer *l* with $X_{l}\in {\mathbb{R}}^{H_{l}^{i}\times W_{l}^{i}\times C_{l}^{i}\times D_{l}^{i}}$ as the input activation tensor, and $W_{l}\in {\mathbb{R}}^{k_{l}^{x}\times k_{l}^{y}\times k_{l}^{z}\times C_{l}^{i}\times C_{l}^{o}}$ as the weight tensor. Assuming no spatial reduction, the total number of floating point operations (FLOP) and memory accesses (Mem), which involves fetching the input activation (IA) tensor, weight (W) tensor, and writing to the output activation (OA) tensor, in layer *l* are given as


(4) $$\begin{array}{l} FLOP_{3D}^{l}=k_{l}^{x}\times k_{l}^{y}\times k_{l}^{z}\times C_{l}^{i}\times C_{l}^{o}\times H_{l}^{i}\times W_{l}^{i}\times D_{l}^{i} \end{array}$$

(5) $$\begin{array}{l} Mem_{3D}^{l}=H_{l}^{i}\times W_{l}^{i}\times C_{l}^{i}\times D_{l}^{i}+k_{l}^{x}\times k_{l}^{y}\times k_{l}^{z}\times C_{l}^{i}\times C_{l}^{o}\\ +H_{l}^{i}\times W_{l}^{i}\times C_{l}^{o}\times D_{l}^{i} \end{array}$$

where the first, second and third term in $Mem_{3D}^{l}$ correspond to IA, W, and OA, respectively. Note that we assume the whole operation can be performed in a single compute substrate (e.g., systolic array), without having to incur any additional data movement, and that the number of operations is independent of activation and weight bit-widths. Similarly, for a 2D CNN layer *l*, the total number of MACs and memory accesses is


(6) $$\begin{array}{l} FLOP_{2D}^{l}=k_{l}^{x}\times k_{l}^{y}\times C_{l}^{i}\times C_{l}^{o}\times H_{l}^{i}\times W_{l}^{i} \end{array}$$

(7) $$\begin{array}{l} Mem_{2D}^{l}=H_{l}^{i}\times W_{l}^{i}\times C_{l}^{i}+k_{l}^{x}\times k_{l}^{y}\times C_{l}^{i}\times C_{l}^{o}+H_{l}^{i}\times W_{l}^{i}\times C_{l}^{o} \end{array}$$

where we do not have the third dimension *D*. From Equations (3–6),


(8) $$\begin{array}{l} \frac{FLOP_{3D}^{l}}{FLOP_{2D}^{l}}=k_{l}^{z}\times D_{l}^{i} \end{array}$$

(9) $$\begin{array}{l} \frac{Mem_{3D}^{l}}{Mem_{2D}^{l}}=\frac{\left(H_{l}^{i}\times W_{l}^{i}\times C_{l}^{i}\times D_{l}^{i}\right)+\left(k_{l}^{x}\times k_{l}^{y}\times k_{l}^{z}\times C_{l}^{i}\times C_{l}^{o}\right)+\left(H_{l}^{i}\times W_{l}^{i}\times C_{l}^{o}\times D_{l}^{i}\right)}{\left(H_{l}^{i}\times W_{l}^{i}\times C_{l}^{i}\right)+\left(k_{l}^{x}\times k_{l}^{y}\times C_{l}^{i}\times C_{l}^{o}\right)+\left(H_{l}^{i}\times W_{l}^{i}\times C_{l}^{o}\right)} \end{array}$$

(10) $$\begin{array}{l} \leq \frac{H_{l}^{i}\times W_{l}^{i}\times C_{l}^{i}\times D_{l}^{i}}{H_{l}^{i}\times W_{l}^{i}\times C_{l}^{i}}+\frac{\left(k_{l}^{x}\times k_{l}^{y}\times k_{l}^{z}\times C_{l}^{i}\times C_{l}^{o}\right)}{\left(k_{l}^{x}\times k_{l}^{y}\times C_{l}^{i}\times C_{l}^{o}\right)}\\ +\frac{\left(H_{l}^{i}\times W_{l}^{i}\times C_{l}^{o}\times D_{l}^{i}\right)}{\left(H_{l}^{i}\times W_{l}^{i}\times C_{l}^{o}\right)} \end{array}$$

(11) $$\begin{array}{l} \leq 2D_{l}^{i}+k_{l}^{z} \end{array}$$

Assuming $k_{l}^{z}=3$ (all SOTA CNN architectures have filter size 3 in each dimension),


(12) $$\begin{array}{l} \frac{FLOP_{3D}^{l}}{FLOP_{2D}^{l}}\geq \frac{Mem_{3D}^{l}}{Mem_{2D}^{l}}\;\text{if }D_{l}^{i}\geq 3 \end{array}$$

Hence, 3D CNNs have higher arithmetic intensity, compared to 2D CNNs, when the spatial dimension *D* is higher than 3. This holds true in all but the last layer of a deep CNN network. For a 100 × 100 input activation tensor with 64 and 128 input and output channels, respectively, adding a third dimension of size 100 (typical hyperspectral images has 100s of spectral bands), and necessitating the use of 3D CNNs, increases the FLOP count by 300×, whereas the memory access cost increases by 96.5×. Note that these improvement factors are obtained by setting the input and output activation dimensions above in Eqs. 8 and 9 and assuming $k_{l}^{x}=k_{l}^{y}=k_{l}^{z}=3$.

Moreover, as shown in Section 7, the energy consumption of a 3D CNN is compute bound on both general-purpose and neuromorphic hardware, and the large increment in FLOPs translates to significant SNN savings in total energy, as an AC operation is significantly cheaper than a MAC operation. Note that SNNs cannot reduce the memory access cost involving the weights.

## 4. Proposed Quantized SNN Training Method

In this section, we evaluate and compare the different choices for SNN quantization in terms of compute efficiency and model accuracy. We then incorporate the chosen quantization technique into STDB, which we refer to as Q-STDB.

### 4.1. Study of Quantization Choice

Uniform quantization transforms a weight element *w* ∈ [*w*_*min*_, *w*_*max*_] to a range [−2^*b*−1^, 2^*b*−1^ − 1] where *b* is the bit-width of the quantized integer representation. There are primarily two choices for the above transformation, known as *affine* and *scale* quantization. In affine quantization, the quantized value can be written as *w*_*a*_ = *s*_*a*_ · *w* + *z*_*a*_, where *s*_*a*_ and *z*_*a*_ denote the scale and zero point (the quantized value to which the real value zero is mapped), respectively. However, scale quantization performs range mapping with only a scale transformation, does not have a zero correction term, and has a symmetric representable range [−α, +α]. Hence, affine quantization leads to more accurate representations compared to the scale counterpart. Detailed descriptions of these two types of quantization can be found in Jain et al. (2020) and Wu et al. (2020).

To evaluate the compute cost of our quantization framework, let us consider a 3D convolutional layer *l*, the dominant layer in HSI classification models, that performs a tensor operation *O*_*l*_ = *X*_*l*_ ⊛ *W*_*l*_ where $X_{l}\in {\mathbb{R}}^{H_{l}^{i}\times W_{l}^{i}\times C_{l}^{i}\times D_{l}^{i}}$ is the IA tensor, $W_{l}\in {\mathbb{R}}^{k_{l}^{x}\times k_{l}^{y}\times k_{l}^{z}\times C_{l}^{i}\times C_{l}^{o}}$ is the W tensor and $O^{l}\in {\mathbb{R}}^{H_{l}^{o}\times W_{l}^{o}\times C_{l}^{o}\times D_{l}^{o}}$ is the OA tensor, with the same notations as used in Section 3. The result of the real-valued operation *O*_*l*_ = *X*_*l*_ ⊛ *W*_*l*_ can be approximated with quantized tensors $X_{l}^{Q}$ and $W_{l}^{Q}$, by first dequantizing them producing $\hat{X_{l}}$ and $\hat{W_{l}}$, respectively, and then performing the convolution. Note that the same quantization parameters are shared by all elements in the weight tensor, because this reduces the computational cost compared to other granularity choices with no impact on model accuracy. Activations are similarly quantized, but only in the input layer, since they are binary spikes in the remaining layers. Also, note that both $X_{l}^{Q}$ and $W_{l}^{Q}$ have similar dimensions as *X*_*l*_ and *W*_*l*_, respectively. Assuming the tensors are scale-quantized per layer,


(13) $$\begin{array}{l} O_{l}=X_{l}⊛W_{l}\approx \hat{X_{l}}⊛\hat{W_{l}}=X_{l}^{Q}⊛W_{l}^{Q}\cdot \left(\frac{1}{s_{s}^{X}\cdot s_{s}^{W}}\right) \end{array}$$

where $s_{s}^{X}$ and $s_{s}^{W}$ are scalar values for scale quantization representing the levels of the input and weight tensor, respectively. Hence, scale quantization results in an integer convolution, followed by a point-wise floating-point multiplication for each output element. Given that a typical 3D convolution operation involves a few thousands of MAC operations (accumulate for binary spike inputs) to compute an output element, a single floating-point operation for the scaling shown in Equation (13) is a negligible computational cost. This is because computing *X*^*l*^ ⊛ *W*^*l*^ involves element-wise multiplications of the weight kernels across multiple channels (for example, for a 3D convolution with 3 × 3 × 3 kernel and 100 channels, we need to perform 2700 MACs) and the corresponding overlapping input activation maps. The accumulated output then needs to be divided by $s_{s}^{X}\cdot s_{s}^{W}$, which adds negligible compute cost.

Although both affine and scale quantization enable the use of low-precision arithmetic, affine quantization results in more computationally expensive inference as shown below.


(14) $$\begin{array}{l} O_{l}\approx \frac{X_{l}^{Q}-z_{a}^{X}}{s_{a}^{X}}⊛\frac{W_{l}^{Q}-z_{a}^{W}}{s_{a}^{W}}\\ =\frac{\left(X_{l}^{Q}⊛W_{l}^{Q}-z_{a}^{X}⊛\left(W_{l}^{Q}-z_{a}^{W}\right)-X_{l}^{Q}⊛z_{a}^{W}\right)}{s_{a}^{X}\cdot s_{a}^{W}} \end{array}$$

Note that $z_{a}^{X}$ and $z_{a}^{W}$ are tensors of sizes equal to that of $X_{l}^{Q}$ and $W_{l}^{Q}$, respectively, that consist of repeated elements of the scalar zero-values of the input activation and weight tensor, respectively. On the other hand, $s_{a}^{X}$ and $s_{a}^{W}$ are the corresponding scale values. The first term in the numerator of Equation (14) is the integer convolution operation similar to the one performed in scale quantization shown in Equation (13). The second term contains integer weights and zero-points, which can be computed offline, and adds an element-wise addition during inference. The third term, however, involves point-wise multiplication with the quantized activation $X_{l}^{Q}$, which cannot be computed before-hand. As we show in Section 7.5.1, this extra computation can increase the energy consumption of our SNN models by over an order of magnitude.

However, our experiments detailed in Section 7 show that ignoring the affine shift during SNN training degrades the test accuracy significantly. Hence, the forward path computations during SNN training follows affine quantization as per (Equation 14), while the other steps involved in SNN training (detailed in Section 4.2), namely gradient computation, and parameter update, use the full-precision weights and membrane potentials, similar to binary ANN training to aid convergence (Courbariaux et al., 2016).

After training, the full-precision weights are rescaled for inference using scale quantization, as per Equation (13), which our results show yields negligible accuracy drop compared to using affine-scaled weights. The membrane potentials obtained as results of the accumulate operations only need to be compared with the threshold voltage once for each time step, which consumes negligible energy, and can be performed using fixed-point comparators (in the periphery of the memory array for PIM accelerators).

Notice that the affine quantization acts as an intermediate representation that lies between full-precision and scale quantization during training; using full-precision causes a large mismatch between weight representations during training and inference, while scale quantization during training results in a similar mismatch during its forward and backward computations. Thus, in principle, this approach is similar to incremental quantization approaches (Zhou et al., 2017) in which we incrementally adjust the type of quantization from the more accurate affine form to more energy-efficient scale form. Lastly, we note that our approach to quantization is also applicable to standard 3D CNNs but the relative savings is significantly higher in SNNs due to the fact that inference is implemented without multiply accumulates.

### 4.2. Q-STDB Based Training

**Figure 3:**
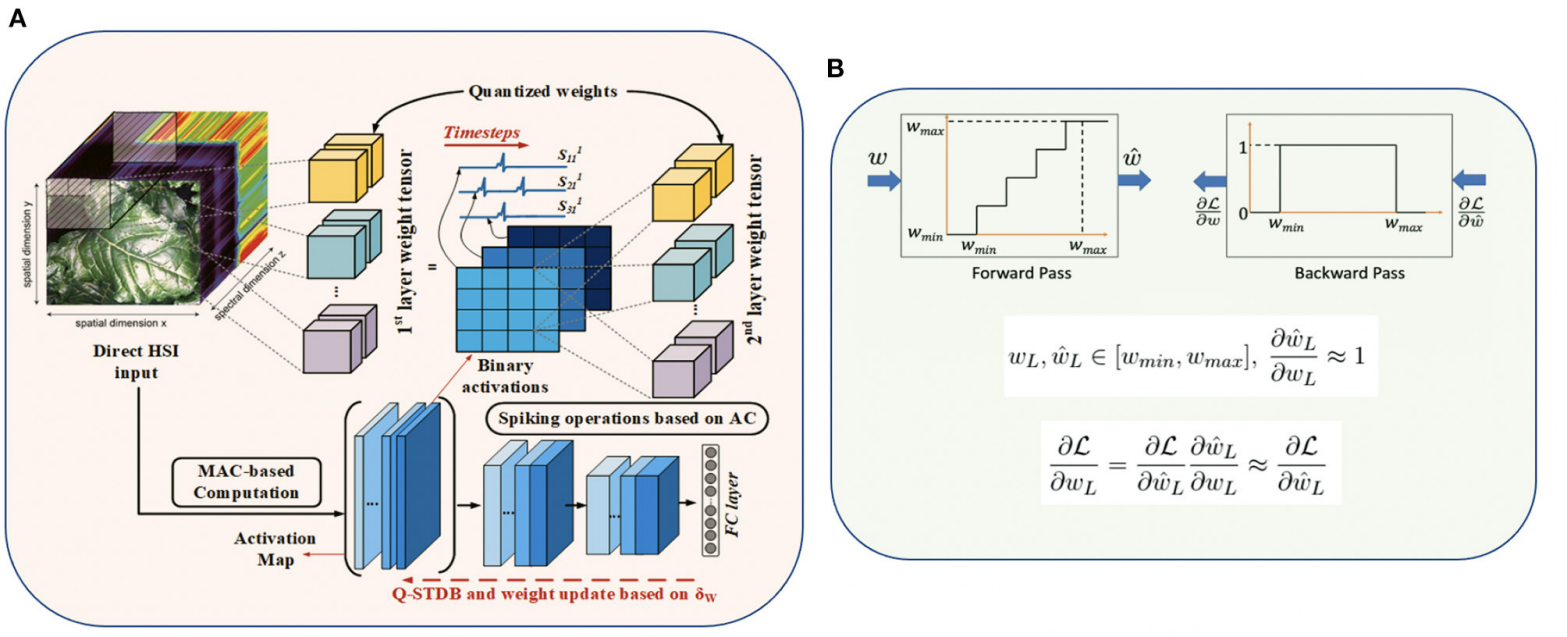
**(A)** Proposed SNN training framework details with 3D convolutions, and **(B)** Fake quantization forward and backward pass with straight through estimator (STE) approximation.

Our proposed training algorithm, illustrated in Figure 3, incorporates the above quantization methodology into the STDB technique (Rathi and Roy, 2020), where the spatial and temporal credit assignment is performed by unrolling the SNN network in time and employing BPTT.

*Output Layer:* The neuron model in the output layer *L* only accumulates the incoming inputs without any leakage, does not generate an output spike, and is described by


(15) $$\begin{array}{l} u_{L}^{t}=u_{L}^{t-1}+\hat{w_{L}}o_{L-1}^{t} \end{array}$$

where *N* is the number of output labels, ***u***_*L*_ is a vector containing the membrane potential of *N* output neurons, ***ŵ***_*L*_ is the affine quantized weight matrix connecting the last two layers (*L* and *L* − 1), and ***o***_*L*−1_ is a vector containing the spike signals from layer (*L* − 1). The loss function is defined on ***u***_*L*_ at the last time step *T* ($u_{L}^{T}$). Since ${\text{u}}_{L}^{T}$ is a vector consisting of continuous values, we compute the SNN's predicted distribution (*p*) as the softmax of ${\text{u}}_{L}^{T}$, similar to the output fully-connected layer of a CNN. Since our SNN is used only for *classification* tasks, we employ the popular cross-entropy loss. The loss function L is thus defined as the cross-entropy between the true one-hot encoded output (*y*) and the distribution *p*.


(16) $$\begin{array}{l} L=-\overset{N}{\underset{i=1}{\sum }}y_{i}log\left(p_{i}\right),\;p_{i}=\frac{e^{u_{i}^{T}}}{\sum_{j=1}^{N}e^{u_{j}^{T}}}, \end{array}$$

The derivative of the loss function with respect to the membrane potential of the neurons in the final layer is described by $\frac{\partial L}{\partial u_{L}^{T}}=\left(p-y\right)$, where ***p*** and ***y*** are vectors containing the softmax and one-hot encoded values of the true label, respectively. To compute the gradient at the current time step, the membrane potential at the previous step is considered as an input quantity (Rathi and Roy, 2020). With the affine-quantized weights in the forward path, gradient descent updates the network parameters ***w***_*L*_ of the output layer as


(17) $$\begin{array}{l} w_{L}=w_{L}-\eta \Delta w_{L} \end{array}$$

(18) $$\begin{array}{l} \Delta w_{L}=\underset{t}{\sum }\frac{\partial L}{\partial w_{L}}=\underset{t}{\sum }\frac{\partial L}{\partial u_{L}^{t}}\frac{\partial u_{L}^{t}}{\partial {ŵ}_{L}}\frac{\partial {ŵ}_{L}}{\partial w_{L}}\\ =\frac{\partial L}{\partial u_{L}^{T}}\underset{t}{\sum }\frac{\partial u_{L}^{t}}{\partial {ŵ}_{L}}\frac{\partial {ŵ}_{L}}{\partial w_{L}}\approx \left(p-y\right)\underset{t}{\sum }o_{L-1}^{t} \end{array}$$

(19) $$\begin{array}{l} \frac{\partial L}{\partial o_{L-1}^{t}}=\frac{\partial L}{\partial u_{L}^{t}}\frac{\partial u_{L}^{t}}{\partial o_{L-1}^{t}}=\left(p-y\right){ŵ}_{L} \end{array}$$

where η is the learning rate (LR). Note that the derivative of the affine quantization function of the weights ($\frac{\partial {ŵ}_{L}}{\partial w_{L}}$) is undefined at the step boundaries and zero everywhere, as shown in Figure 3A. Our training framework addresses this challenge by using the Straight-through Estimator (STE) (Courbariaux et al., 2016), which approximates the derivative to be equal to 1 for inputs in the range [*w*_*min*_, *w*_*max*_] as shown in Figure 3B, where *w*_*min*_ and *w*_*max*_ are the minimum and maximum values of the weights in a particular layer. Note that *w*_*min*_ and *w*_*max*_ are updated at the end of every mini-batch to ensure all the weights lie between *w*_*min*_ and *w*_*max*_ during the forward and backward computations in each training iteration. Hence, we use $\frac{\partial {ŵ}_{L}}{\partial w_{L}}\approx 1$ to compute the loss gradients in Equation (18).

*Hidden layers*: The neurons in all the hidden layers follow the quantized LIF model shown in Equation (2). All neurons in a layer possess the identical leak and threshold value. This reduces the number of trainable parameters and we did not observe any noticeable accuracy change by assigning different threshold/leak value to each neuron, similar to Datta et al. (2021). With a single threshold for each layer, it may seem redundant to train both the weights and threshold together. However, we observe, similar to Rathi and Roy (2020) and Datta et al. (2021) that the latency required to obtain the SOTA classification accuracy decreases with the joint optimization, which further drops by training the leak term. This may be because the loss optimizer can reach an improved local minimum when all the parameters are tunable. The weight update in Q-STDB is calculated as


(20) $$\begin{array}{l} \Delta w_{l}=\underset{t}{\sum }\frac{\partial L}{\partial w_{l}}=\underset{t}{\sum }\frac{\partial L}{\partial z_{l}^{t}}\frac{\partial z_{l}^{t}}{\partial o_{l}^{t}}\frac{\partial o_{l}^{t}}{\partial u_{l}^{t}}\frac{\partial u_{l}^{t}}{\partial {ŵ}_{l}}\frac{\partial {ŵ}_{l}}{\partial w_{l}}\\ \approx \underset{t}{\sum }\frac{\partial L}{\partial z_{l}^{t}}\frac{\partial z_{l}^{t}}{\partial o_{l}^{t}}\frac{1}{v_{l}}o_{l-1}^{t}\cdot 1 \end{array}$$

where $\frac{\partial {ŵ}_{l}}{\partial w_{l}}$ and $\frac{\partial z_{l}^{t}}{\partial o_{l}^{t}}$ are the two discontinuous gradients. We calculate the former using STE described above, while the latter is approximated using surrogate gradient (Bellec et al., 2018) shown below.


(21) $$\begin{array}{l} \frac{\partial z_{l}^{t}}{\partial o_{l}^{t}}=\gamma \cdot max\left(0,1-|z_{l}^{t}|\right) \end{array}$$

Note that γ is a hyperparameter denoting the maximum value of the gradient. The threshold and leak update is computed similarly using BPTT (Rathi and Roy, 2020).

## 5. SRAM-Based PIM Acceleration

Efficient hardware implementations of neural network algorithms are being widely explored by the research community in an effort to enable intelligent computations on resource constrained edge devices (Chen et al., 2020). Existing computing systems based on the well-known von-Neumann architecture (characterized by physically separated memory and computing units) suffer from energy and throughput bottleneck, referred as the *memory wall bottleneck* (Agrawal et al., 2018; Dong et al., 2018). Novel memory-centric paradigms like PIM are being extensively investigated by the research community to mitigate the energy-throughput constraints arising from the memory wall bottleneck. As discussed in Section 1, the first layer of a direct coded SNN is not as computationally efficient as the other layers, as it processes continuous valued inputs as opposed to spiking inputs, and dominates the total energy consumption. Further, for 3D images such as HSI, the number of real valued computations in the first layer of an SNN is orders of magnitude more than 2D images.

**Figure 4:**
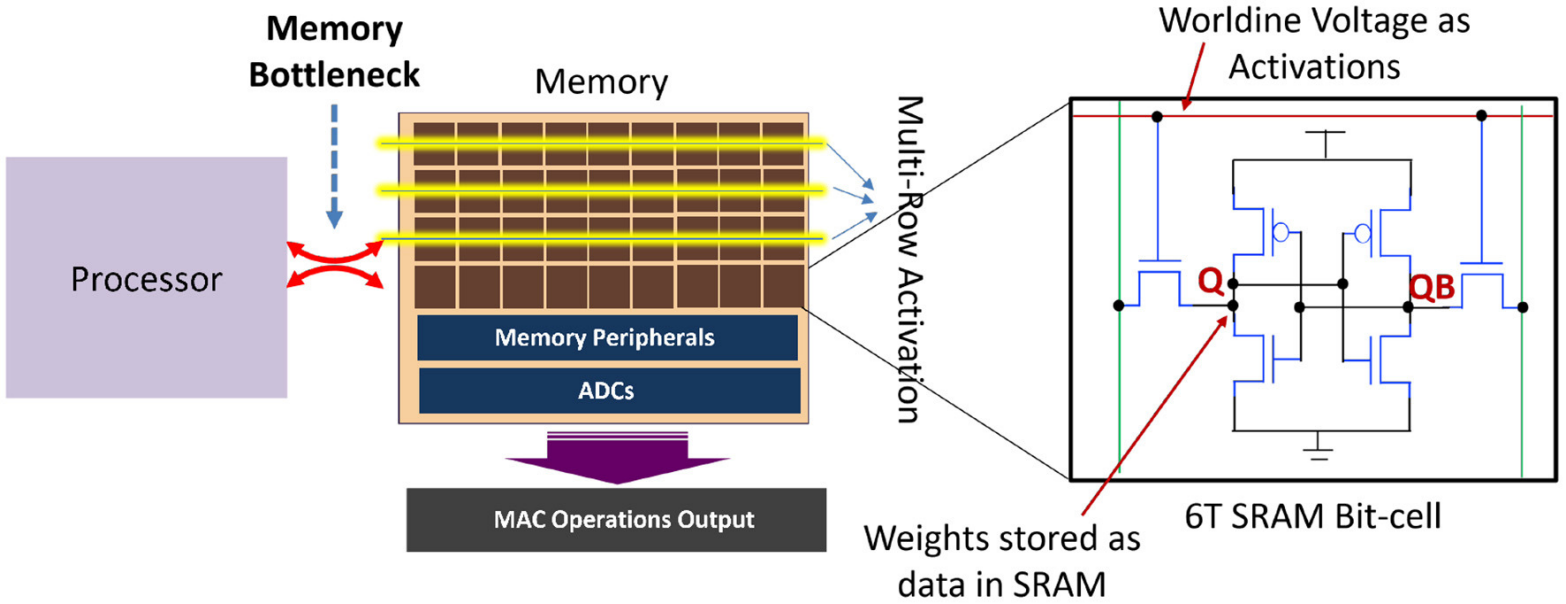
PIM architecture in the first layer to process MAC operations for the first layer of direct coded SNNs. Other layers of the SNN are processed with highly parallel programmable architecture using simpler accumulate operations.

In order to enable energy-efficient hardware for SNNs catering to 3D images, we propose to exploit the high-parallelism, high-throughput and low-energy benefits of analog PIM in SRAM, for the first layer of the SNN. As mentioned earlier, the first layer of SNN requires real valued MAC operations which are well-suited to be accelerated using analog PIM approaches (Kang et al., 2018; Ali et al., 2020). Moreover, the number of weights in the first layer of a typical 3D CNN architecture is substantially less compared to the other layers, which ensures that we can perform PIM using a single memory array, thereby reducing the complexity of the peripheral circuits, such as adder trees for partial sum reduction. Several proposals achieving multiple degrees of compute parallelism within on-chip memory based on SRAM arrays have been proposed (Agrawal et al., 2018, 2019; Dong et al., 2018; Biswas and Chandrakasan, 2019; Jaiswal et al., 2019). Interestingly, both digital (Agrawal et al., 2018; Dong et al., 2018) as well as analog- mixed-signal approaches (Agrawal et al., 2019; Biswas and Chandrakasan, 2019) have been explored extensively. Analog approaches are of particular importance due to higher levels of data parallelism and compute throughput compared to digital counterparts in performing MAC computations. Our adopted PIM architecture for the first layer of our proposed SNNs is illustrated in Figure 4. The PIM architecture leverages analog computing for parallel MAC operations by mapping activations as voltages on the wordlines and weights as data stored in the SRAM bit-cells (represented as Q and QB). As shown in Ali et al. (2020), multi-bit MAC operations can be enabled in SRAM arrays by activating multiple rows, simultaneously, allowing appropriately weighted voltages to develop on each column of the SRAM array representing the resulting MAC operations computed in analog domain. Peripheral ADC circuits are used to convert the analog MAC operation into corresponding digital data for further computations.

To summarize, we propose use of analog PIM to accelerate the MAC intensive compute requirements for the first layer of the SNN. The remaining layers of the SNN leverage traditional digital hardware implementing simpler accumulate operations. Advantageously, our proposed quantized SNN with small number of weights in the first layer is well-suited for low-overhead PIM circuits, as reduction in bit-precision and peripheral complexity drastically improves the energy and throughput efficiency of analog PIM architectures (Kang et al., 2018).

## 6. Proposed CNN Architectures, Datasets, and Training Details

### 6.1. Model Architectures

We developed two models, a 3D and a hybrid fusion of 3D and 2D convolutional architectures, that are inspired by the recently proposed CNN models (Ben Hamida et al., 2018; Luo et al., 2018; Roy et al., 2020) used for HSI classification and compatible with our ANN-SNN conversion framework. We refer to the two models CNN-3D and CNN-32H.

**Figure 5:**
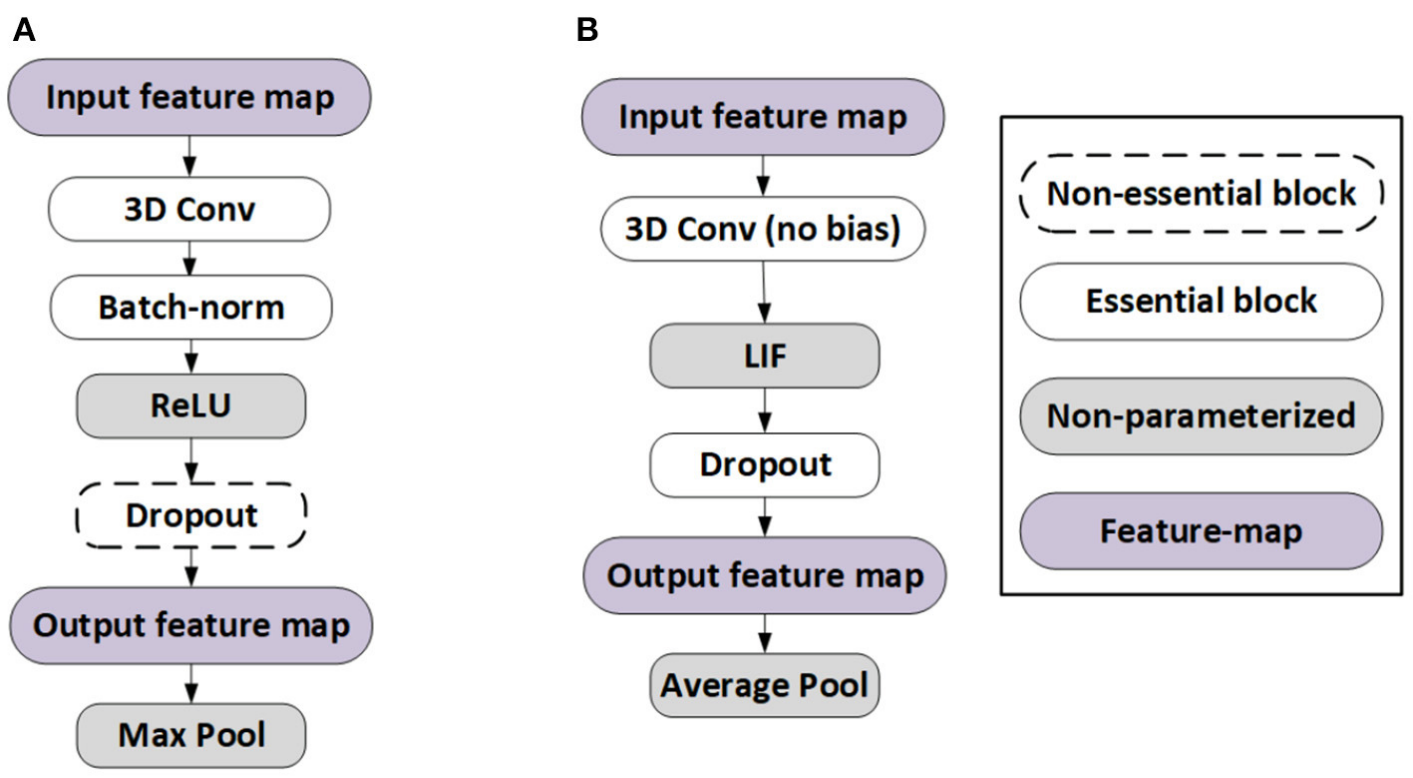
Architectural differences between **(A)** ANN and **(B)** SNN for near-lossless ANN-SNN conversion.

There are several constraints in the training of the baseline ANN models needed to obtain near lossless ANN-SNN conversion (Diehl et al., 2016; Sengupta et al., 2019). In particular, we omit the bias term from the ANN models because the integration of the bias term over multiple SNN timesteps tends to shift the activation values away from zero which causes problems in the ANN-SNN conversion process (Sengupta et al., 2019). In addition, similar to Sengupta et al. (2019), Rathi and Roy (2020), Rathi et al. (2020), and Kim and Panda (2021), we do not use batch normalization (BN) layers because using identical BN parameters (e.g., global mean μ, global standard deviation σ, and trainable parameter γ) for the statistics of all timesteps do not capture the temporal dynamics of the spike train in an SNN. Instead, we use dropout (Srivastava et al., 2014) as the regularizer for both ANN and SNN training. Recent research (Rathi and Roy, 2020; Rathi et al., 2020) indicates that there is no problem in yielding state-of-the-art accuracy in complex image recognition tasks, such as CIFAR-100, with models without batch normalization and bias. We observe the same for HSI models in this work as well. Moreover, our initial ANN models employ ReLU nonlinearity after each convolutional and linear layer (except the classifier layer), due to the similarity between ReLU and LIF neurons. Our pooling operations use average pooling because for binary spike based activation layers, max pooling incurs significant information loss. Our SNN-specific architectural modifications are illustrated in Figure 5.

We also modified the number of channels and convolutional layers to obtain compact yet accurate models. 2D patches of sizes 5 × 5 and 3 × 3 were extracted for CNN-3D and CNN-32H, respectively, without any reduction in dimensionality from each dataset. Higher sized patches increase the computational complexity without any significant improvement in test accuracy. Note that magnitude based structured weight pruning (Han et al., 2015b), which has been shown to be an effective technique for model compression, can only remove <15% of the weights averaging across the two architectures, with <1% degradation in test accuracy for all the three datasets used in our experiments, which also indicates the compactness of our models. The details of both models are given in Table 1.

**Table 1:** Model architectures employed for CNN-3D and CNN-32H in classifying the IP dataset.

**Layer**	**Size of input**	**Number of**	**Size of**	**Stride**	**Padding**	**Dropout**	**Size of output**
**type**	**feature map**	**filters**	**each filter**	**value**	**value**	**value**	**feature map**
**Architecture : CNN-3D**							
3D Convolution	(5,5,200,1)	20	(3,3,3)	(1,1,1)	(0,0,0)	-	(3,3,198,20)
3D Convolution	(3,3,198,20)	40	(1,1,3)	(1,1,2)	(1,0,0)	-	(3,3,99,40)
3D Convolution	(3,3,99,40)	84	(3,3,3)	(1,1,1)	(1,0,0)	-	(1,1,99,84)
3D Convolution	(1,1,99,84)	84	(1,1,3)	(1,1,2)	(1,0,0)	-	(1,1,50,84)
3D Convolution	(1,1,50,84)	84	(1,1,3)	(1,1,1)	(1,0,0)	-	(1,1,50,84)
3D Convolution	(1,1,50,84)	84	(1,1,2)	(1,1,2)	(1,0,0)	-	(1,1,26,84)
**Architecture : CNN-32H**							
3D Convolution	(3,3,200,1)	90	(3,3,18)	(1,1,7)	(0,0,0)	-	(1,1,27,90)
2D Convolution	(27,90,1)	64	(3,3)	(1,1)	(0,0)	-	(25,88,64)
2D Convolution	(25,88,64)	128	(3,3)	(1,1)	(0,0)	-	(23,86,128)
Avg. Pooling	(23,86,128)	-	(4,4)	(4,4)	(0,0)	-	(59,21,128)
Dropout	(5,21,128)	-	-	-	-	0.2	(5,21,128)
Linear	13,440	6,881,280	-	-	-	-	512

*Every convolutional and linear layer is followed by a ReLU non-linearity. The last classifier layer is not shown. The size of the activation map of a 3D CNN is written as (H,W,D,C) where H, W, D, and C represent the height, width, depth of the input feature map and the number of channels. Since the 2D CNN layer does not have the depth dimension, its feature map size is represented as (H,W,C)*.

### 6.2. Datasets

**Figure 6:**
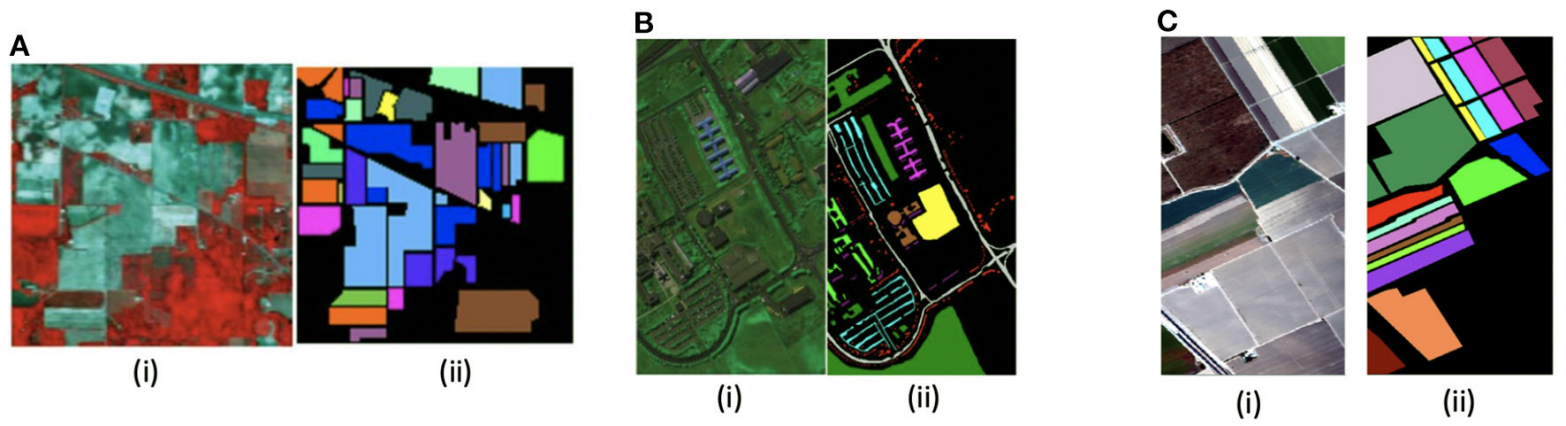
(i) False color-map and (ii) ground truth images of different HSI datasets used in our work, namely **(A)** Indian Pines, **(B)** Pavia University, and **(C)** Salinas Scene.

We used four publicly available datasets, namely Indian Pines, Pavia University, Salinas scene, and HyRANK. A brief description follows for each one, and few sample images found in some of these datasets are shown in Figure 6.

*Indian Pines*: The Indian Pines (IP) dataset consists of 145 × 145 spatial pixels and 220 spectral bands in a range of 400–2,500 nm. It was captured using the AVIRIS sensor over North-Western Indiana, USA, with a ground sample distance (GSD) of 20 m and has 16 vegetation classes.

*Pavia University*: The Pavia University (PU) dataset consists of hyperspectral images with 610 × 340 pixels in the spatial dimension, and 103 spectral bands, ranging from 430 to 860 nm in wavelength. It was captured with the ROSIS sensor with GSD of 1.3 m over the University of Pavia, Italy. It has a total of 9 urban land-cover classes.

*Salinas Scene*: The Salinas Scene (SA) dataset contains images with 512 × 217 spatial dimension and 224 spectral bands in the wavelength range of 360–2, 500 nm. The 20 water absorbing spectral bands have been discarded. It was captured with the AVIRIS sensor over Salinas Valley, California with a GSD of 3.7 m. In total 16 classes are present in this dataset.

*HyRANK*: The ISPRS HyRANK dataset is a recently released hyperspectral benchmark dataset. Different from the above HSI datasets that contain a singlr hyperspectral scene, the HyRANK dataset consists of two hyperspectral scenes, namely Dioni and Loukia. Similar to Meng et al. (2021), we use the available labeled samples in the Dioni scene for training, while those in the Loukia scene for testing. The Dioni and Loukia scenes comprise 250 × 1, 376 and 249 × 945 spectral samples, respectively, and each has 176 spectral reflectance bands.

For preprocessing, images in all the data sets are normalized to have a zero mean and unit variance. For our experiments, all the samples (except that of the HyRANK dataset) are randomly divided into two disjoint training and test sets. The limited 40% samples are used for training and the remaining 60% for performance evaluation.

### 6.3. ANN Training and SNN Conversion Procedures

We start by performing full-precision 32-bit ANN training for 100 epochs using the standard SGD optimizer with an initial learning rate (LR) of 0.01 that decayed by a factor of 0.1 after 60, 80, and 90 epochs.

The ANN-SNN conversion entails the estimation of the values of the weights and per-layer thresholds of the SNN model architecture. The weights are simply copied from a trained DNN model to the iso-architecture target SNN model. The threshold for each layer is computed sequentially as the 99.7 percentile of the pre-activation distribution (weighted sum of inputs received by each neuron in a layer) over the total number of timesteps (Rathi and Roy, 2020) for a small batch of HSI images (of size 50 in our case). Note that we use 100 time steps to evaluate the thresholds, while the SNN training and inference are performed with only 5 time steps. In our experiments we scale the initial layer thresholds by 0.8. We keep the leak of each layer set to unity while evaluating these thresholds. Note that employing direct coding as used in our work and others (Rathi and Roy, 2020) can help avoid any approximation error arising from the input spike generation (conversion from raw images to spike trains) process and aid ANN-SNN conversion. Lower bit-precision of weights will most likely not exacerbate the conversion process, assuming the ANN models can be trained accurately with the same bit-precision.

We then perform quantization-aware SNN training as described in Section 4 for another 100 epochs. We set γ = 0.3 (Bellec et al., 2018) and used the ADAM optimizer with a starting LR of 10^−4^ which decays by a factor of 0.5 after 60, 80, and 90 epochs. All experiments are performed on a Nvidia 2080Ti GPU with 11 GB memory.

## 7. Experimental Results and Analysis

This section first describes our inference accuracy results, then analyzes the associated spiking and energy consumption. It then describes several ablation studies and a comparison of the training time and memory requirements.

### 7.1. ANN and SNN Inference Results

**Figure 7:**
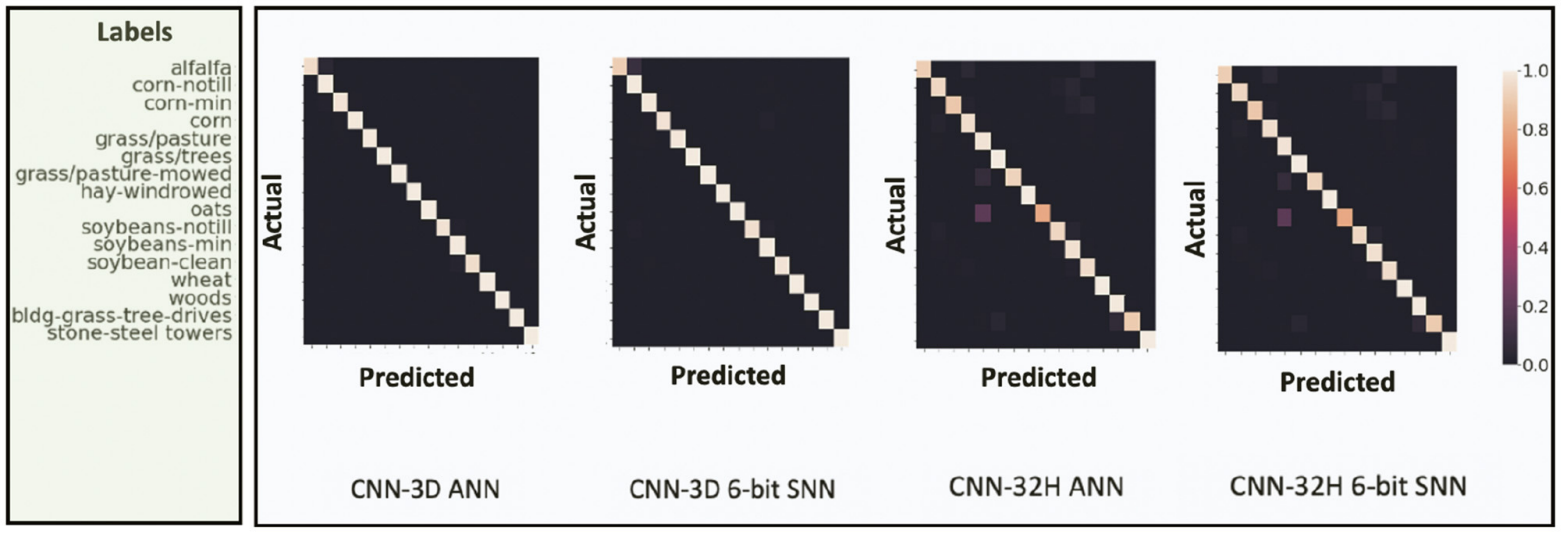
Confusion Matrix for HSI test performance of ANN and proposed 6-bit SNN over IP dataset for both CNN-3D and CNN-32H. The ANN and SNN confusion matrices look similar for both the network architectures. CNN-32H incurs a little drop in accuracy compared to CNN-3D due to shallow architecture.

We report the best Overall Accuracy (OA), Average Accuracy (AA), and Kappa Coefficient measures to evaluate the HSI classification performance for our proposed architectures, similar to Ben Hamida et al. (2018). Here, OA represents the number of correctly classified samples out of the total test samples. AA represents the average of class-wise classification accuracies, and Kappa is a statistical metric used to assess the mutual agreement between the ground truth and classification maps. Column-2 in Table 2 shows the ANN accuracies, column-3 shows the accuracy after ANN-SNN conversion with 50 time steps^1^. Column-4 shows the accuracy when we perform our proposed training without quantization, while columns 5 to 7 shows the SNN test accuracies obtained with Q-STDB for different weight bit precisions (4 to 6 bits). SNNs trained with 6-bit weights result in 5.33× reduction in bit-precision compared to full-precision (32-bit) models and, for all three tested data sets, perform similar to the full precision ANNs for both the CNN-3D and CNN-32H architectures. Although the membrane potentials do not need to be quantized as described in Section 4, we observed that the model accuracy does not drop significantly even if we quantize them, and hence, the SNN results shown in Table 2 correspond to 6-bit membrane potentials. Four-bit weights and potentials provide even lower complexity, but at the cost of a small accuracy drop. Figure 7 shows the confusion matrix for the HSI classification performance of the ANN and proposed SNN over the IP dataset for both the architectures.

**Table 2:** Model performances with Q-STDB based training on IP, PU, SS, and HyRANK datasets for CNN-3D and CNN-32H after (A) ANN training, (B) ANN-to-SNN conversion, (C) 32-bit SNN training, (D) 4-bit SNN training, (E) 5-bit SNN training, and (F) 6-bit SNN training, with only 5 time steps.

	**A. ANN**			**B. Accuracy after**			**C. Accuracy after**			**D. Accuracy after**			**E. Accuracy after**			**F. Accuracy after**		
**Dataset**	**accuracy (** ***%*** **)**			**ANN-to-SNN conv. (** ***%*** **)**			**FP SNN training (** ***%*** **)**			**4-bit SNN training (%)**			**5-bit SNN training (%)**			**6-bit SNN training (%)**		
	**OA**	**AA**	**Kappa**	**OA**	**AA**	**Kappa**	**OA**	**AA**	**Kappa**	**OA**	**AA**	**Kappa**	**OA**	**AA**	**Kappa**	**OA**	**AA**	**Kappa**
**Architecture : CNN-3D**																		
IP	98.86	98.42	98.55	57.68	50.88	52.88	98.92	98.76	98.80	97.08	95.64	95.56	98.38	97.78	98.03	98.68	98.34	98.20
PU	99.69	99.42	99.58	91.16	88.84	89.03	99.47	99.06	99.30	98.21	97.54	97.75	99.26	98.48	98.77	99.50	99.18	99.33
SS	98.89	98.47	98.70	81.44	76.72	80.07	98.49	97.84	98.06	96.47	93.16	94.58	97.25	95.03	95.58	97.95	97.09	97.43
HyRANK	64.21	63.27	47.34	34.80	58.97	20.64	63.18	61.25	45.25	59.76	56.40	42.28	61.70	60.48	46.06	62.96	61.27	46.82
**Architecture : CNN-32H**																		
IP	97.60	97.08	97.44	70.88	66.56	67.89	97.27	96.29	96.35	96.63	95.81	95.89	97.23	96.08	96.56	97.45	96.73	96.89
PU	99.50	99.09	99.30	94.96	90.12	93.82	99.38	98.83	99.13	99.17	98.41	98.68	99.25	98.84	98.86	99.35	98.88	98.95
SS	98.88	98.39	98.67	88.16	84.19	85.28	97.92	97.20	97.34	97.34	96.32	96.77	97.65	96.81	96.97	97.99	97.26	97.38
HyRANK	64.43	70.68	52.82	24.26	26.90	19.37	63.72	67.89	49.59	62.27	62.50	46.58	63.27	65.32	47.98	63.34	66.66	48.21

The inference accuracy (OA, AA, and Kappa) of our ANNs and SNNs trained via Q-STDB are compared with the current state-of-the-art ANNs used for HSI classification in Table 3. As we can see, simply porting the ANN architectures used in Ben Hamida et al. (2018) and Luo et al. (2018) to SNNs, and performing 6-bit Q-STDB results in significant drops in accuracy, particularly for the India Pines data set. In contrast, our CNN-3D-based SNN models suffer negligible OA drop (<1% for all datasets) compared to the best performing ANN models for HSI classification.

**Table 3:** Inference accuracy (OA, AA, and Kappa) comparison of our proposed SNN models obtained from CNN-3D and CNN-32H with state-of-the-art deep ANNs on IP, PU, SS, and HyRANK datasets.

**References**	**ANN/SNN**	**Architecture**	**OA (*%*)**	**AA (*%*)**	**Kappa (*%*)**
**Dataset : Indian Pines**					
Alipour-Fard et al. (2020)	ANN	MSKNet	81.73	71.4	79.2
Song et al. (2018)	ANN	DFFN	98.52	97.69	98.32
Zhong et al. (2018)	ANN	SSRN	99.19	98.93	99.07
Roy et al. (2020)	ANN	HybridSN	**99.75**	**99.63**	**99.71**
Ben Hamida et al. (2018)	ANN	6-layer 3D CNN	98.29	97.52	97.72
	SNN		95.88	94.26	95.34
Luo et al. (2018)	ANN	Hybrid CNN	96.15	94.96	95.73
	SNN		94.90	94.08	94.78
This work	ANN	CNN-3D	98.86	98.42	98.55
	SNN		98.79	98.34	98.60
This work	ANN	CNN-32H	97.60	97.08	97.44
	SNN		97.45	96.73	96.89
**Dataset : Pavia University**					
Alipour-Fard et al. (2020)	ANN	MSKNet	90.66	88.09	87.64
Song et al. (2018)	ANN	DFFN	98.73	97.24	98.31
Zhong et al. (2018)	ANN	SSRN	99.61	**99.56**	99.33
Meng et al. (2021)	ANN	DRIN	96.4	95.8	95.2
Ben Hamida et al. (2018)	ANN	6-layer 3D CNN	99.32	99.02	99.09
	SNN		98.55	98.02	98.28
Luo et al. (2018)	ANN	Hybrid CNN	99.05	98.35	98.80
	SNN		98.40	97.66	98.21
This work	ANN	CNN-3D	**99.69**	99.42	**99.58**
	SNN		99.50	99.18	99.33
This work	ANN	CNN-32H	99.50	99.09	99.30
	SNN		99.35	98.88	98.95
**Dataset : Salinas Scene**					
Song et al. (2018)	ANN	DFFN	98.87	**98.75**	98.63
Meng et al. (2021)	ANN	DRIN	96.7	98.6	96.3
Luo et al. (2018)	ANN	Hybrid CNN	98.85	98.35	98.22
	SNN		97.05	97.41	97.18
This work	ANN	CNN-3D	**98.89**	98.47	**98.70**
	SNN		97.95	97.09	97.43
This work	ANN	CNN-32H	98.88	98.39	98.67
	SNN		97.99	97.26	97.38
**Dataset : HyRANK**					
Meng et al. (2021)	ANN	DRIN	54.4	56.0	43.3
This work	ANN	CNN-3D	64.21	63.27	47.34
	SNN		62.96	61.27	46.82
This work	ANN	CNN-32H	**64.43**	**69.68**	**52.82**
	SNN		63.34	66.66	48.21

*The bold values indicate maximum values*.

### 7.2. Spiking Activity

Each SNN spike involves a constant number of AC operations, and hence, consumes a fixed amount of energy. Consequently, the average spike count of an SNN layer *l*, denoted ζ_*l*_, can be treated as a measure of compute-energy of the model (Sengupta et al., 2019; Rathi et al., 2020). We calculate ζ_*l*_ as the ratio of the total spike count in *T* steps over all the neurons of layer *l* to the number of neurons in the layer. Hence, the energy efficiency of an SNN model can be improved by decreasing the spike count.

**Figure 8:**
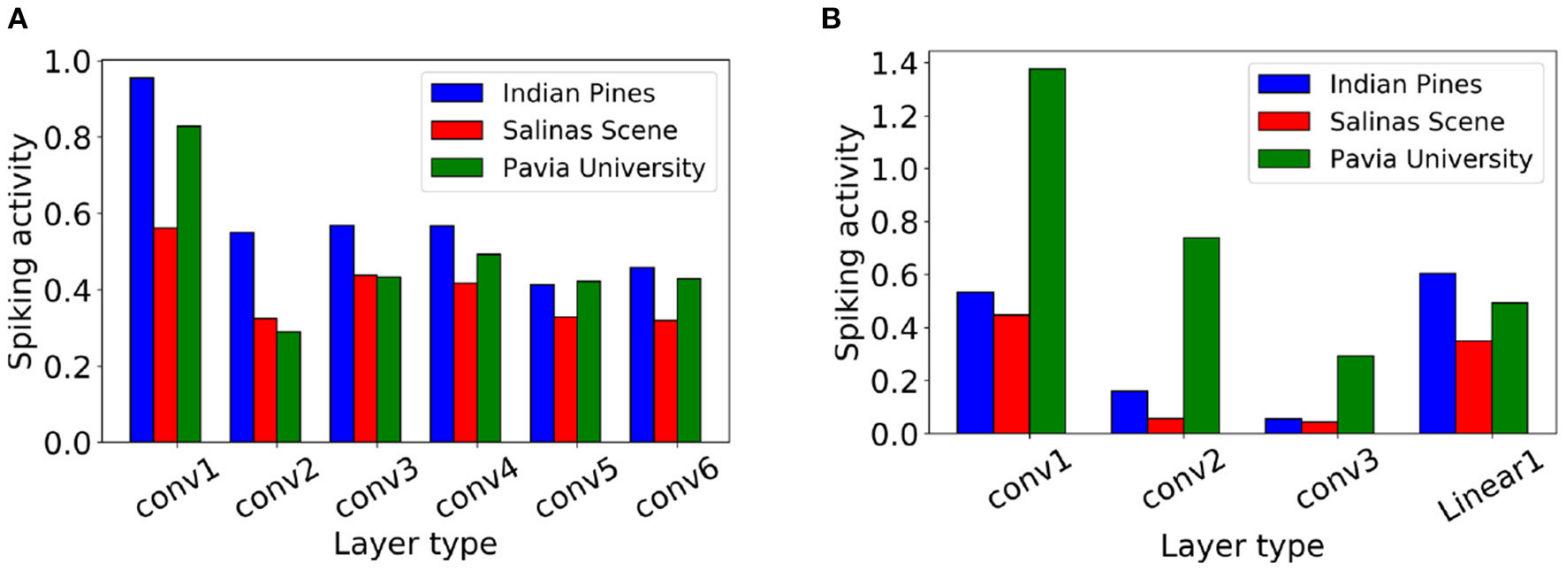
Layerwise spiking activity plots for **(A)** CNN-3D and **(B)** CNN-32H on Indian Pines, Salinas Scene and Pavia University datasets.

Figure 8 shows the average spike count for each layer with Q-STDB when evaluated for 200 samples from each of the three datasets (IP, PU, SS) for the CNN-3D and CNN-32H architecture. For example, the average spike count of the 3^*rd*^ convolutional layer of the CNN-3D-based SNN for IP dataset is 0.568, which means each neuron in that layer spikes 0.568 times on average over all input samples over a 5 time step period. Note that the average spike count is less than 1.4 for all the datasets across both the architectures which leads to significant energy savings as described below.

### 7.3. Energy Consumption and Delay

In this section, we analyze the improvements in energy, delay, and EDP of our proposed SNN models compared to the baseline SOTA ANN models running on digital hardware for all the three datasets. We show that further energy savings can be obtained by using the PIM architecture discussed in Section 5 to process the first layer of our SNN models.

#### 7.3.1. Digital Hardware

Let us assume a 3D convolutional layer *l* having weight tensor ${\text{W}}^{l}\in {\mathbb{R}}^{k\times k\times k\times C_{l}^{i}\times C_{l}^{o}}$ that operates on an input activation tensor ${\text{I}}^{l}\in {\mathbb{R}}^{H_{l}^{i}\times W_{l}^{i}\times C_{l}^{i}\times D_{l}^{i}}$, where the notations are similar to the one used in Section 4. We now quantify the energy consumed to produce the corresponding output activation tensor ${\text{O}}^{l}\in {\mathbb{R}}^{H_{l}^{o}\times W_{l}^{o}\times C_{l}^{o}\times D_{l}^{o}}$ for an ANN and SNN, respectively. Our model can be extended to fully-connected layers with $f_{l}^{i}$ and $f_{l}^{o}$ as the number of input and output features, respectively, and to 2D convolutional layers, by shrinking a dimension of the feature maps.

In particular, for any layer *l*, we extend the energy model of Ali et al. (2020) and Kang et al. (2018) to 3D CNNs by adding the third dimension of weights (*k*) and output feature maps ($D_{l}^{o}$), as follows


(22) $$\begin{array}{c} E_{l}^{CNN}=C_{l}^{i}C_{l}^{o}k^{3}E_{read}+C_{l}^{i}C_{l}^{o}k^{3}H_{l}^{o}W_{l}^{o}D_{l}^{o}E_{mac}+P_{leak}T_{l}^{CNN} \end{array}$$

where the first term denotes the memory access energy, the second term denotes the compute energy, while the third term highlights the static leakage energy. Note that *T*_*l*_ is the latency incurred to process the layer *l*, and can be written as


(23) $$\begin{array}{c} T_{l}^{CNN}=\left(\frac{C_{l}^{i}C_{l}^{o}k^{3}}{\frac{B_{IO}}{B_{W}}N_{bank}}\right)T_{read}+\left(\frac{C_{l}^{i}C_{l}^{o}k^{3}}{N_{mac}}\right)H_{l}^{o}W_{l}^{o}D_{l}^{o}T_{mac} \end{array}$$

The notations for Equations (22) and (23), along with their values, obtained from Kang et al. (2018) and Ali et al. (2020) are illustrated in Table 4. The total energy is compute bound since the compute energy alone consumes ~98% of the total energy averaged across all the layers for the CNN-3D architecture on all the datasets. The memory cost only dominates the few fully connected layers, accounting for >85% of their total energy.

**Table 4:** Notations and their values used in energy, delay, and EDP equations for ANN and 6-bit SNNs.

**Notation**	**Description**	**Value**
*B*_*IO*_	Number of bits fetched from SRAM to processor per bank	64
*B*_*W*_	Bit width of the weight stored in SRAM	6
*N*_*col*_	Number of columns in SRAM array	256
*N*_*bank*_	Number of SRAM banks	4
*N*_*mac*_(*N*_*ac*_)	Number of MACs (ACs) in processing element (PE) array	175 (175)
*T*_*read*_	Time required to transfer 1-bit data between SRAM and PE	4 ns
*T*_*BLP*_	Time required for one analog in-memory accumulation	4 ns
*E*_*mac*_(*E*_*ac*_)	Energy consumed in a single MAC (AC)	3.1 pJ (0.1 pJ) for 32-bit
	Operation for a particular bit-precision	full-precision inputs (Horowitz, 2014)
*T*_*mac*_(*T*_*ac*_)	Time required to perform a single MAC (AC) in PE	4 ns (0.4 ns)
*T*_*adc*_	Time required for a single ADC operation	6 ns
*E*_*read*_	Energy to transfer each weight element between SRAM and PE	5.2 pJ
*E*_*BLP*_	Energy required for a single in-memory analog accumulation	0.08 pJ
*E*_*adc*_	Energy required for an ADC operation	0.268 pJ

Similarly, we can extend the energy and delay model of Kang et al. (2018) and Ali et al. (2020) to our proposed SNNs, as follows


(24) $$\begin{array}{l} E_{l}^{SNN}=C_{l}^{i}C_{l}^{o}k^{3}E_{read}+C_{l}^{i}C_{l}^{o}k^{3}H_{l}^{o}W_{l}^{o}D_{l}^{o}\zeta_{l}E_{ac}+P_{leak}T_{l}^{SNN} \end{array}$$

(25) $$\begin{array}{l} T_{l}^{SNN}=\left(\frac{C_{l}^{i}C_{l}^{o}k^{3}}{\frac{B_{IO}}{B_{W}}N_{bank}}\right)T_{read}+\left(\frac{C_{l}^{i}C_{l}^{o}k^{3}}{N_{ac}}\right)H_{l}^{o}W_{l}^{o}D_{l}^{o}T_{ac} \end{array}$$

for any layer *l* except the input layer that is based on direct encoding, whose energy and delay can be obtained from Equations (22, 23), respectively. The notations used in Equations (23, 24), along with their values are also shown in Table 4. Notice that the spiking energy in Equation (22) assume the use of zero-gating logic that activates the compute unit only when an input spike is received and thus is a function of spiking activity ζ_*l*_. However, to extend the benefits of a low ζ^*l*^ to latency, we require either custom hardware or compiler support (Liu et al., 2018). For this reason, unlike energy, this paper assumes no delay benefit from ζ_*l*_ as is evident in Equation (25).

To compute *E*_*MAC*_ for full-precision weights (full-precision and 6-bits) and *E*_*AC*_ (6-bits) at 65 nm technology, we use the data from Horowitz (2014) obtained by silicon measurements (see Table 4). For 6-bit inputs, we scale the energy according to $E_{mac}\propto Q^{1.25}$ as shown in Moons et al. (2017), where *Q* is the bit-precision. On the other hand, *E*_*ac*_ (6-bits) is computed by scaling the full-precision data from Horowitz (2014), according to Simon et al. (2019), which shows *E*_*AC*_ is directly proportional to the data bit-width. Our calculations imply that *E*_*AC*_ is ~13× smaller than *E*_*MAC*_ for 6-bit precision. Note that this number may vary for different technologies, but, in most technologies, an AC operation is significantly less expensive than a MAC operation. As required in the direct input encoding layer, we obtain *E*_*mac*_ for 8-bit inputs and 6-bit weights from Kang et al. (2018), applying voltage scaling for iso-*V*_*dd*_ conditions with the other *E*_*mac*_ and *E*_*ac*_ estimations from Horowitz (2014). We use *T*_*ac*_ = 0.1*T*_*mac*_ for 6-bit inputs from Ganesan (2015) and the fact that the latency of a MAC unit varies logarithmically with bit precision (assuming a carry-save adder) to calculate the delay, and the resulting EDP of the baseline SOTA ANN and our proposed SNN models. Note that the architectural modifications applied to the existing SOTA models to create our baseline ANNs (Ben Hamida et al., 2018; Roy et al., 2020) only enhance ANN-SNN conversion, and do not lead to significant changes in energy consumption. Since the total energy is compute bound, we also calculate the total number of floating point operations (FLOPs), which is a standard metric to evaluate the energy cost of ML models.

**Figure 9:**
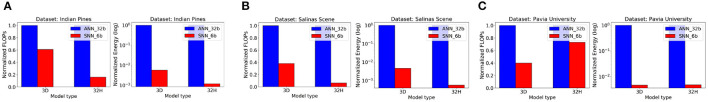
Comparison of FLOPs and compute energy of CNN-3D and CNN-32H between ANN and SNN models while classifying on **(A)** Indian Pines, **(B)** Salinas Scene, and **(C)** Pavia University datasets, respectively.

**Figure 10:**
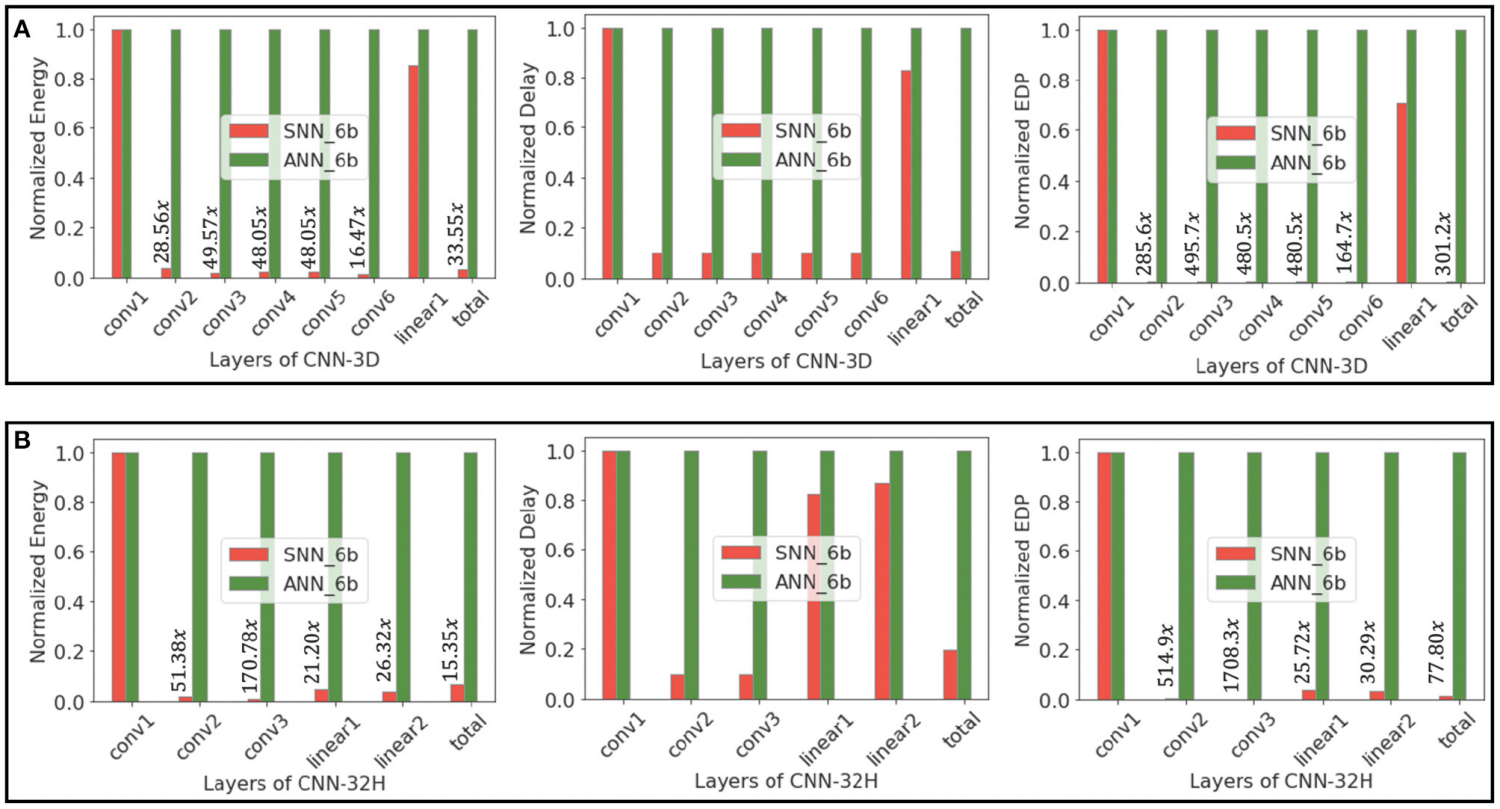
Energy, delay, and EDP of layers of **(A)** CNN-3D and **(B)** CNN-32H architectures, comparing 6-bit ANNs and SNN (obtained via Q-STDB) models while classifying IP.

Figure 9 illustrates the total energy consumption and FLOPs for full precision ANN and 6-bit quantized SNN models of the two proposed architectures, where the energy is normalized to that of the baseline ANN. We also consider 6-bit ANN models to compare the energy efficiency of low-precision ANNs and SNNs. We observe that 6-bit ANN models are 12.5× energy efficient compared to 32-bit ANN models due to significant improvements in MAC energy with quantization, as shown in Moons et al. (2017). Note that we can achieve similar HSI test accuracies shown in Table 2 with quantized ANNs as well. We compare the layer-wise and total energy, delay, and EDP of our proposed SNNs with those of equivalent-precision ANNs in Figure 10.

The FLOPs for SNNs obtained by our proposed training framework is smaller than that for the baseline ANN due to low spiking activity. Moreover, because the ACs consume significantly less energy than MACs for all bit precisions, SNNs are significantly more compute efficient. In particular, for CNN-3D on IP, our proposed SNN consumes ~199.3× and ~33.8× less energy than an iso-architecture full-precision and 6-bit ANN with similar parameters, respectively. The improvements become ~560.6× (~9976× in EDP) and ~44.8× (~412.2× in EDP), respectively, averaging across the two network architectures and three datasets.

#### 7.3.2. PIM Hardware

Though SNNs improve the total energy significantly as shown above, the first layer needs the expensive MACs due to direct encoding, and accounts for ~27% and ~22% of the total energy on average across the three datasets for CNN-3D and CNN-32H, respectively. To address this issue, we propose to adopt an SRAM-based memory array to process the computations incurred in the first layer, in the memory array itself, as discussed in Section 5.

We similarly extended the energy and delay models of Ali et al. (2020) and Kang et al. (2018) to the PIM implementation of the first layer of our proposed SNN architectures. The resulting energy and delay can be written as


(26) $$\begin{array}{l} E_{1}^{SNN}=C_{1}^{i}C_{1}^{o}k^{3}\left(E_{BLP}+\frac{E_{ADC}}{R}\right)+P_{leak}T_{1}^{SNN} \end{array}$$

(27) $$\begin{array}{l} T_{1}^{SNN}=\left(\frac{C_{1}^{i}C_{1}^{o}k^{3}}{\frac{N_{col}}{B_{W}}N_{bank}}\right)H_{1}^{o}W_{1}^{o}D_{1}^{o}\left(T_{read}+\frac{T_{adc}}{R}\right) \end{array}$$

where the new notations along with their values are in Table 4. Following 65 nm CMOS technology limitations, we keep the array parameters similar to Kang et al. (2018), and *T*_*adc*_ and *E*_*adc*_ for our 6-bit SNN are obtained by extending the circuit simulation results of Ali et al. (2020) with the ADC energy and delay models proposed in Gonugondla et al. (2020).

**Figure 11:**
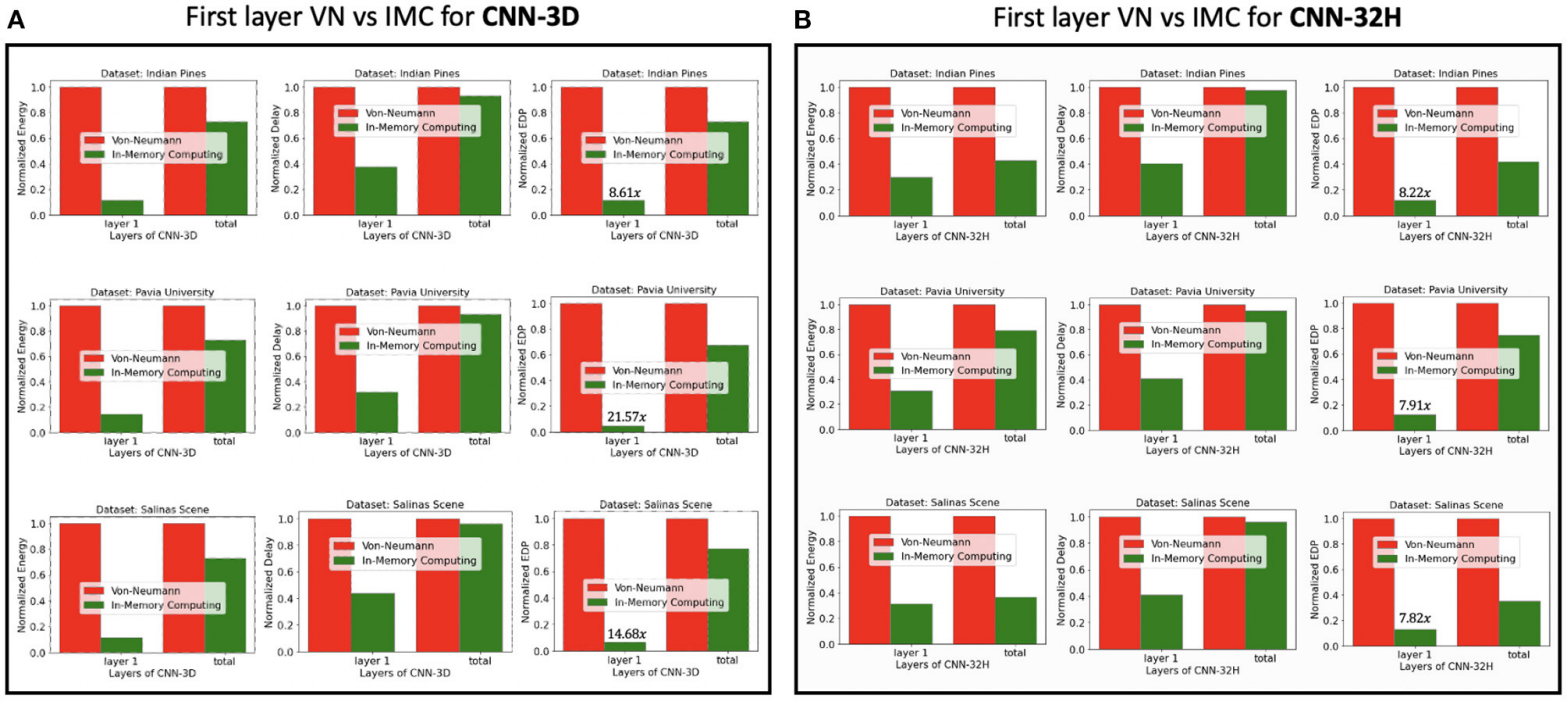
Energy, delay, and EDP comparison of traditional digital and in-memory computing (only 1^*st*^ layer) hardware for the SNN models obtained with **(A)** CNN-3D, and **(B)** CNN-32H architectures classifying Indian Pines, Pavia University, and Salinas Scene datasets.

Figure 11 compares the energy, delay and the EDP of the first-layer-PIM implementation of the spiking version of CNN-3D and CNN-32H against the corresponding digital implementations for the IP, PU, and SS datasets. The improvements in the total energy, delay and EDP for CNN-3D on IP dataset, are seen to be 1.28×, 1.08× and 1.38×, respectively, over an iso-architecture-and-precision SNN implemented with digital hardware. The improvements become 1.30×, 1.07× and 1.38×, respectively, averaging across the three datasets. However, since CNN-32H is shallower than CNN-3D, and has relatively cheaper 2D CNNs following the input 3D CNN layer, the PIM implementation in the first layer can decrease the total energy consumption significantly. The energy, delay, and EDP improvements compared to the digital implementations are estimated to be 2.12×, 1.04×, and 2.20× for CNN-32H, and 1.71×, 1.06×, and 1.79× on average across the two architectures and three datasets. Hence, the total improvements for our proposed hybrid hardware implementation (PIM in first layer and digital computing in others), coupled with our energy-aware quantization and training technique, become 953×, 17.76×, 16921× compared to iso-architecture full-precision ANNs and 76.16×, 9.2×, 700.7× compared to iso-architecture iso-precision ANNs.

Note that analog-PIM based SNNs are more cheaper in terms of energy consumption than their CNN counterparts. This is because of the reasons summarized below.

- Since CNN requires both multi-bit activations and multi-bit weights, the precision of ADCs and DACs required in analog-PIM based CNN accelerator is higher than for analog-SNN based accelerators. As is well known, ADCs are the most energy-expensive components in analog PIM accelerators, thus, this higher precision requirement leads to higher energy consumption. For example, an 8 bit ADC consumes 2× more energy compared to a 4 bit ADC (Ali et al., 2021).
- The limited precision of ADCs also necessitates ‘bit-streaming’ (Ankit et al., 2020), wherein multi-bit activations of CNN are serially streamed to analog-PIM crossbars and accumulated over time. Such serial streaming increases both delay and power consumption for computing.
- Finally, the higher algorithmic sparsity associated with SNN leads to reduction in energy consumption while performing analog-PIM operations. Note that this sparsity can also be leveraged by custom digital hardware.

However, the energy-delay benefit associated with analog-PIM based SNNs with respect to digital SNN implementation is lower as compared to analog-PIM based CNN in comparison digital CNN implementation. This is because CNNs require extensive energy-hungry multiplication operations, while SNNs rely on cheaper accumulate operations. Moreover, analog PIM implementation leads to increased non-idealities and can decrease the resulting test accuracy of our HSI models. As the number of weights increases after the first layer (4.5× in the 2^*nd*^ layer to 352.8× in the 6th layer for CNN-3D), a single layer has to be mapped over multiple memory sub-arrays. This, in turn, requires partial sums generated from individual sub-arrays to be transferred via Network-on-chip (NoC) for accumulation and generation of output activation. The NoC and associated data transfer incurs increase in energy-delay and design complexity. Hence, we choose to avoid PIM in the subsequent layers.

### 7.4. Training Time and Memory Requirements

**Figure 12:**
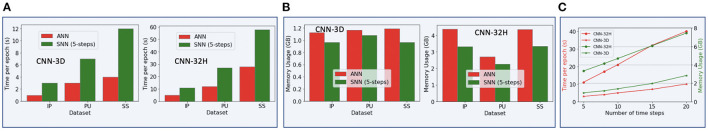
Comparison between our baseline SOTA ANNs and proposed SNNs with 5 time steps based on **(A)** training time per epoch, and **(B)** memory usage during training. Variation of **(A,B)** with the number of time steps for the IP dataset and CNN-32H architecture are shown in **(C)**.

We also compared the simulation time and memory requirements during the training of the baseline SOTA ANN and our proposed SNN models. Because SNNs require iterating over multiple time steps and storing the membrane potentials for each neuron, their simulation time and memory requirements can be substantially higher than their ANN counterparts. However, training with ultra low-latency, as done in this work, can bridge this gap significantly as shown in Figure 12. We compare the simulation time and memory usage during training of the baseline ANNs and our proposed SNN models in Figures 12A,B, respectively. As we can see, the training time per epoch is less than a minute for all the architectures and datasets. Moreover, the peak memory usage during training is also lower for our SNN models compared to their ANN counterparts. Hence, we conclude that our approach does not incur any significant training overhead. Note that both the training time and memory usage are higher for CNN-32H than for CNN-3D because the output feature map of its last convolutional layer is very large.

### 7.5. Ablation Studies

We conducted several ablation studies on combinations of affine and scale quantization during training and inference, quantized training approaches, and the efficacy of ANN-based pre-training.

#### 7.5.1. Affine vs. Scale Quantization

**Figure 13:**
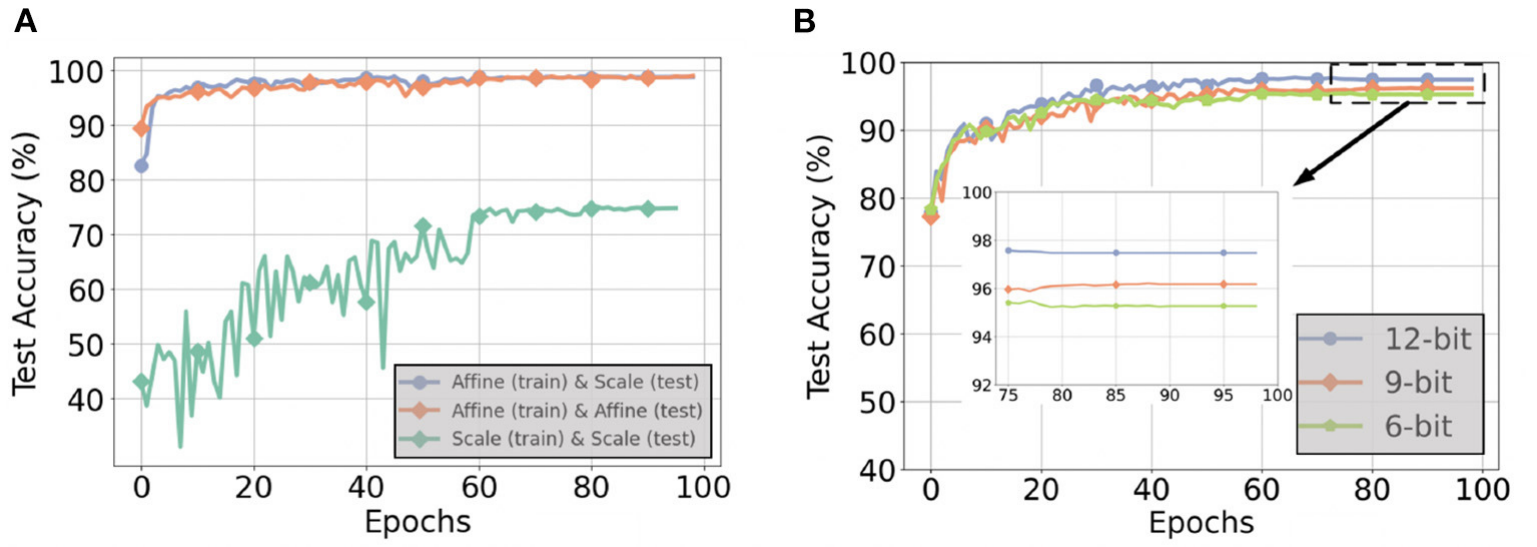
**(A)** Test accuracies for different quantization techniques during the forward path of training and inference with a 6-bit CNN-3D model on the IP dataset with 5 timesteps, **(B)** Test accuracies with 6, 9, and 12-bit weight precisions for post-training quantization with a CNN-32H model on the IP dataset with 5 timesteps.

Figure 13A compares inference accuracies for three different quantization techniques during the forward path of training and test on the CNN-3D architecture with the IP dataset using 6-bit quantization. Performing scale quantization during training significantly degrades performance, which further justifies our use of affine quantization during training. However, using scale quantization during inference results in similar accuracy as affine quantization. We further explored the gap in accuracy for 4-bit and 5-bit quantization, as summarized in Table 5. We observed that the accuracy gap associated with using scale quantization instead of affine quantization during inference modestly grows to 1.42% for 4-bit weights.

**Table 5:** Loss in accuracy associated with use of scale quantization during inference.

	**A. Affine (training) and**			**B. Affine (training) and**		
**Bit-precision**	**Affine (inference)**			**Scale (inference)**, Δ **from Column A**.		
	**OA (*%*)**	**AA (*%*)**	**Kappa (*%*)**	**Δ OA (*%*)**	**Δ AA (*%*)**	**Δ Kappa (*%*)**
6	98.89	98.39	98.21	0.21	0.05	0.01
5	98.79	98.36	98.24	0.41	0.13	0.21
4	98.50	98.01	98.07	1.42	2.37	2.53

*Evaluated using the CNN-3D model on the IP dataset*.

This small drop in relative accuracy for low bit-precisions may be attributed to the benefit of the zero factor in affine quantization on quantization error. Quantization error is typically measured by half of the width of the quantization bins, where the number of bins *N*_*B*_ used is independent of the type of quantization and, due to the 2's complement representation, centered around zero. However, the range of values these bins must span is smaller for affine quantization because the zero factor ensures the distribution of values is also centered at zero. This difference in range can be calculated as $\Delta =r^{\text{scale}}-r^{\text{affine}}=2\cdot max\left(w_{max},|w_{min}|\right)-\left(w_{max}-w_{min}\right)$. Assuming *w*_*min*_ = −*x*·*w*_*max*_,


(28) $$\begin{array}{c} \Delta \;=\{\begin{array}{ll} (1-x)w_{max}, & \text{if }w_{max}>-w_{min}\\ (x-1)w_{max}, & \text{otherwise}. \end{array} \end{array}$$

**Figure 14:**
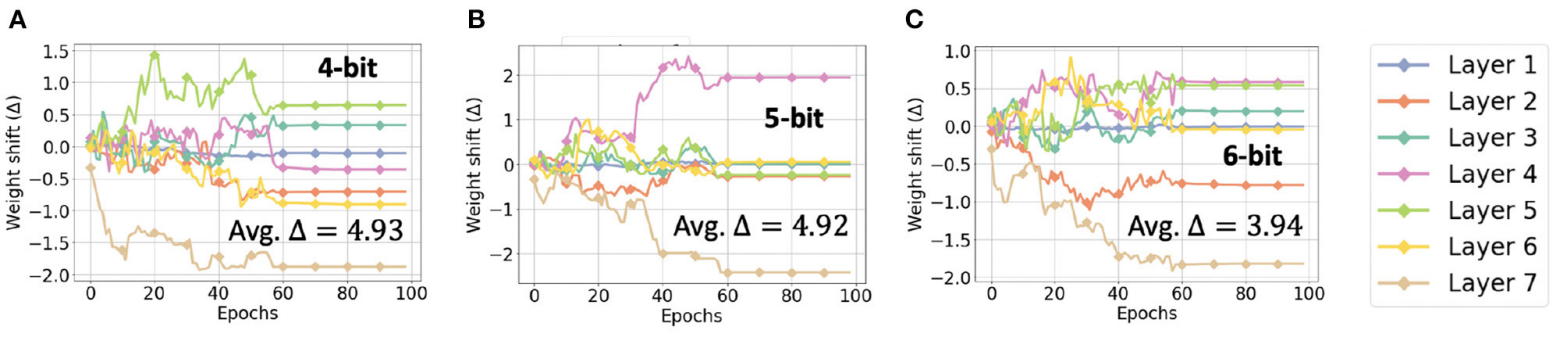
Weight shift (Δ) in each layer of CNN-3D for **(A)** 4, **(B)** 5, and **(C)** 6-bit quantization, while classifying the IP dataset.

As empirically shown in Figure 14, the average Δ across all the layers increases modestly as we decrease the bit-precision from 6 to 4. In contrast, the increase in quantization error associated with scale quantization is equal to $\frac{\Delta }{2N_{B}}$ and thus grows exponentially as the number of bits decrease.

#### 7.5.2. Q-STDB vs. Post-training Quantization

PTQ with scale representation cannot always yield ultra low-precision SNNs with SOTA test accuracy. For example, as illustrated in Figure 13B, for the IP dataset and CNN-32H architecture with 5 time steps, the lowest bit precision of the weights that the SNNs can be trained with PTQ for no more than 1% reduction in SOTA test accuracy is 12, two times larger bit-width than required by Q-STDB. Interestingly, the weights can be further quantized to 8-bits with less than 1% accuracy reduction if we increase the time steps to 10, but this costs latency.

#### 7.5.3. Comparison Between Q-STDB With and Without ANN-SNN Conversion

To quantify the extent that the ANN-based pre-training helps, we performed Q-STDB from scratch (using 5 time steps), where the weights are initialized from the standard Kaiming normal distribution. The results are reported in Table 6, where the results in the columns labeled B and C are obtained by comparing those from the columns labeled F and B, respectively in Table 2 with Q-STDB without ANN-SNN conversion. The results show that while Q-STDB from scratch beats conversion-only approaches, the inference accuracy can often be further improved using our proposed hybrid training combining Q-STDB and ANN-SNN conversion.

**Table 6:** Comparison between model performances for Q-STDB from scratch, proposed hybrid training, and ANN-SNN conversion alone.

		**A. Q-STDB** ***from***			**B. Diff. between proposed hybrid training**			**C. Diff. between ANN-SNN conversion alone**		
**Architecture**	**Dataset**	**scratch**			**and Q-STDB from scratch**			**and Q-STDB from scratch**		
		**OA (*%*)**	**AA (*%*)**	**Kappa (*%*)**	**Δ OA (*%*)**	**Δ AA (*%*)**	**Δ Kappa (*%*)**	**Δ OA (*%*)**	**Δ AA (*%*)**	**Δ Kappa (*%*)**
	IP	96.83	96.25	96.23	1.85	2.11	1.97	-39.15	-45.37	-43.35
CNN-3D	PU	99.38	99.04	99.17	0.14	0.13	0.16	-8.22	-10.2	-10.14
	SS	96.05	95.79	95.60	1.90	1.30	1.83	-14.61	-19.07	-15.53
	IP	95.93	95.36	95.40	1.53	1.37	1.49	-25.05	-28.8	-27.51
CNN-32H	PU	99.12	98.49	98.55	0.23	0.39	0.40	-4.16	-8.37	-4.73
	SS	96.04	95.90	95.33	1.95	1.36	1.95	-7.88	-11.71	-10.05

*All cases are for 5 time steps and 6-bits*.

## 8. Conclusions and Broader Impact

In this paper, we extensively analyse the arithmetic intensities of 3D and 2D CNNs, and motivate the use of energy-efficient, low-latency, LIF-based SNNs for applications involving 3D image recognition, that requires 3D CNNs for accurate processing. We then present a quantization-aware training technique, that yields highly accurate low-precision SNNs. We propose to represent weights during the forward path of training using affine quantization and during the inference forward path using scale quantization. This provides a good trade-off between the SNN accuracy and inference complexity. We propose a 3D and hybrid combination of 3D and 2D convolutional architectures that are compatible with ANN-SNN conversion for HSI classification; the hybrid architecture incurs a small accuracy drop compared to the 3D counterpart, which shows the efficacy of 3D CNNs for HSI. Our quantized SNN models offer significant improvements in energy consumption compared to both full and low-precision ANNs for HSI classification. We also propose a PIM architecture to process the energy-expensive first layer of our direct encoded SNN to further reduce the energy, delay and EDP of the SNN models.

Our proposal results in energy-efficient SNN models that can be more easily deployed in HSI or 3D image sensors and thereby mitigates the bandwidth and privacy concerns associated with off-loading inference to the cloud. This improvement in energy-efficiency is particularly important as the applications of HSI analysis expand and the depth of the SOTA models increases (Boldrini et al., 2012).

To the best of our knowledge, this work is the first to address energy efficiency of HSI models, and can hopefully inspire more research in algorithm-hardware co-design of neural networks for size, weight, and power (SWAP) constrained HSI applications.

## Data Availability Statement

The datasets used for this study can be found in http://www.ehu.eus/ccwintco/index.php?title=Hyperspectral_Remote_Sensing_Scenes. More analysis on the effect of quantization on our proposed models are included in the article/Supplementary Material.

## Author Contributions

GD conceived the idea and performed the simulations required to evaluate the efficacy of SNNs for HSI and prepared the first draft. AJ helped in the analysis of energy, delay, and EDP for the PIM implementation. All authors helped in writing the article. All authors contributed to the article and approved the submitted version.

## Funding

This work was supported in part by the NSF CCF-1763747 award, and in part by the DARPA HR00112190120 award.

## Conflict of Interest

The authors declare that the research was conducted in the absence of any commercial or financial relationships that could be construed as a potential conflict of interest.

## Publisher's Note

All claims expressed in this article are solely those of the authors and do not necessarily represent those of their affiliated organizations, or those of the publisher, the editors and the reviewers. Any product that may be evaluated in this article, or claim that may be made by its manufacturer, is not guaranteed or endorsed by the publisher.

## Footnotes

^1^We empirically observe that at least 50 time steps are required for lossless ANN-SNN conversion.
