# Supplementary material for: ACE-SNN: Algorithm-Hardware Co-design of Energy-Efficient & Low-Latency Deep Spiking Neural Networks for 3D Image Recognition
Source: Front Neurosci. 2022 Apr 7;16:815258. doi: 10.3389/fnins.2022.815258 (PMC9025538; doi:10.3389/fnins.2022.815258)
Supplement: Supplementary file 1 [file Data_Sheet_1.PDF]

Variation of Model Accuracies with Bit-Precision

1 SUPPLEMENTARY DATA

Table S1. Model performances with Q-STDB based training on IP, PU, SS, and HyRANK datasets for CNN-3D and CNN-32H after a) Full-precision SNN training, b) 6-bit SNN training, c) 7-bit SNN training, d) 8-bit SNN training, and e) 9-bit SNN training, with only **5** time steps.

Dataset	A. Accuracy after FP SNN training (%)			B. Accuracy after **6**-bit SNN training (%)			C. Accuracy after **7**-bit SNN training (%)			D. Accuracy after **8**-bit SNN training (%)			E. Accuracy after **9**-bit SNN training (%)		
	OA	AA	Kappa	OA	AA	Kappa	OA	AA	Kappa	OA	AA	Kappa	OA	AA	Kappa
Architecture : CNN-3D															
IP	98.92	98.76	98.80	98.68	98.34	98.20	98.63	98.50	98.43	98.79	98.46	98.75	98.79	98.60	98.83
PU	99.47	99.06	99.30	99.50	99.18	99.33	99.46	99.09	99.34	99.50	99.09	99.21	99.48	99.16	99.29
SS	98.49	97.84	98.06	97.95	97.09	97.43	97.94	97.30	97.70	98.22	97.52	98.00	98.09	97.93	97.37
HyRANK	63.18	61.25	45.25	62.96	61.27	46.82	62.98	61.25	46.70	62.78	61.07	45.99	63.10	61.33	45.64
Architecture : CNN-32H															
IP	97.27	96.29	96.35	97.45	96.73	96.89	97.30	96.66	96.39	97.47	96.20	96.73	97.51	96.58	96.60
PU	99.38	98.83	99.13	99.35	98.88	98.95	99.35	98.74	99.24	99.40	98.85	99.05	99.37	98.87	99.21
SS	97.92	97.20	97.34	97.99	97.26	97.38	97.73	97.22	97.34	97.90	97.14	97.53	97.88	97.03	97.09
HyRANK	63.72	67.89	49.59	63.34	66.66	48.21	63.26	66.90	48.34	63.49	67.67	49.02	63.43	67.45	49.40

In the main manuscript, we have showed that the overall accuracies of our proposed SNN models monotonically increase as we increase the bit-precision from 4 to 6 on all the datasets. we have claimed our 6-bit SNN models yield near lossless accuracy compared to the full-precision counterparts, as the lowest bit-precision that yielded accuracies within 1% of the baseline is 6. In particular, the difference in accuracies between the 6-bit and full-precision models is $<0.39\%$ for the HyRANK dataset, $<0.25\%$ for the IP dataset, $<0.03\%$ for the PU dataset, and $<0.55\%$ for the SS dataset. Note that these results are obtained by averaging across three random seeds used to initialize the model parameters. We ran experiments with higher bit-precision and the results are shown in Table S1. The results show that the resulting accuracy improves but with diminishing returns as the bit-width is increased. We note that some of the quantized SNN models in fact yield slightly higher overall accuracy ($<0.16\%$ for the HyRANK dataset) compared to the full-precision counterparts. This might be due to the regularization effect provided by quantization.
